# Genome-Based Comparison of All Species of the Genus *Moorella*, and Status of the Species *Moorella thermoacetica* and *Moorella thermoautotrophica*

**DOI:** 10.3389/fmicb.2019.03070

**Published:** 2020-01-17

**Authors:** Stephanie Redl, Anja Poehlein, Carola Esser, Frank R. Bengelsdorf, Torbjørn Ø. Jensen, Christian B. Jendresen, Brian J. Tindall, Rolf Daniel, Peter Dürre, Alex T. Nielsen

**Affiliations:** ^1^Novo Nordisk Foundation Center for Biosustainability, Technical University of Denmark, Lyngby, Denmark; ^2^Genomic and Applied Microbiology & Göttingen Genomics Laboratory, Georg-August University, Göttingen, Germany; ^3^Institut für Mikrobiologie und Biotechnologie, Universität Ulm, Ulm, Germany; ^4^Leibniz-Institut DSMZ-Deutsche Sammlung von Mikroorganismen und Zellkulturen GmbH, Brunswick, Germany

**Keywords:** anaerobic, thermophile, acetogen, gas fermentation, syngas fermentation, phylogenetic analysis, *Moorella*, *Moorella thermoacetica*

## Abstract

Fermentation of gases provides a promising opportunity for the production of biochemicals from renewable resources, which has resulted in a growing interest in acetogenic bacteria. Thermophilic organisms provide potential advantages for the fermentation of, e.g., syngas into for example volatile compounds, and the thermophiles *Moorella thermoacetica* and *Moorella thermoautotrophica* have become model organisms of acetogenic metabolism. The justification for the recognition of the closely related species *M. thermoautotrophica* has, however, recently been disputed. In order to expand knowledge on the genus, we have here genome sequenced a total of 12 different *M. thermoacetica* and *M. thermoautotrophica* strains. From the sequencing results, it became clear that *M. thermoautotrophica* DSM 1974^T^ consists of at least two different strains. Two different strains were isolated in Lyngby and Ulm from a DSM 1974^T^ culture obtained from the DSMZ (Leibniz-Institut DSMZ-Deutsche Sammlung von Mikroorganismen und Zellkulturen GmbH, Brunswick, Germany). Phylogenetic analysis revealed a close relationship between all the sequenced genomes, suggesting that the two strains detected in the type strain of the species *M. thermoautotrophica* could not be distinguished at the species level from *M. thermoacetica*. Despite genetic similarities, differences in genomic features were observed between the strains. Differences in compounds that can serve as carbon and energy sources for selected strains were also identified. On the contrary, strain DSM 21394, currently still named *M. thermoacetica*, obviously represents a new *Moorella* species. In addition, based on genome analysis and comparison *M. glycerini* NMP, *M. stamsii* DSM 26217^T^, and *M. perchloratireducens* An10 cannot be distinguished at the species level. Thus, this comprehensive analysis provides a significantly increased knowledge of the genetic diversity of *Moorella* strains.

## Introduction

Interest from the research community and industry in acetogenic bacteria has grown within recent years due to their potential to produce valuable compounds from syngas (Latif et al., 2014). Thermophilic acetogens are of significance, since their use would reduce gas cooling requirements, allow for cost-efficient recovery of products with relatively low boiling point (Henstra et al., 2007; Redl et al., 2017), and decrease the risk of contamination.

A well-studied syngas-fermenting thermophile is *Moorella thermoacetica*. The species was isolated from horse feces in 1942 and named *Clostridium thermoaceticum* (Fontaine et al., 1942). The taxonomy of the genus *Clostridium* was restructured in 1994 and *C. thermoaceticum* was transferred to a new genus *Moorella* as *M. thermoacetica* (Collins et al., 1994). Several strains originating from the cultures isolated by Fontaine et al. (1942) are deposited in strain collections. The type strain DSM 521^T^ and the strain ATCC 39073 have primarily served to elucidate the primary metabolism of *M. thermoacetica* (synonym *C. thermoaceticum*): they were used in experiments to study carbohydrate utilization (Andreesen et al., 1973), the acetate kinase (Schaupp and Ljungdahl, 1974), cytochromes and menaquinones (Gottwald et al., 1975), the formate dehydrogenase (Ljungdahl and Andreesen, 1977), and the utilization of CO (Diekert and Thauer, 1978). The genome of the non-type strain ATCC 39073 was sequenced in 2008 (Pierce et al., 2008) and the genome sequence of the type strain DSM 521^T^ followed in 2015 (Poehlein et al., 2015). A spore sample of the original *M. thermoacetica* strain isolated in 1942 was deposited by Kerby and Zeikus (1983) as a second representative of the type strain (DSM 2955^T^) in the DSMZ (Leibniz-Institut DSMZ-Deutsche Sammlung von Mikroorganismen und Zellkulturen GmbH, Brunswick, Germany). It was shown to utilize H_2_/CO_2_ as substrate and was also adapted to growth on CO (Kerby and Zeikus, 1983). The ability to utilize gaseous substrates was not shown for ATCC 39073 and DSM 521^T^ until 1990 (Daniel et al., 1990). Another *M. thermoacetica* strain (Y72) with higher transformation efficiency than ATCC 39073 was described and its draft genome published in 2014 (Tsukahara et al., 2014).

Wiegel et al. (1981) described the isolation of strains closely related to the already known *C. thermoaceticum* (*M. thermoacetica*) strains. The novel strains were shown to grow chemolithotrophically on H_2_/CO_2_ and chemoheterotrophically on several carbon sources. At that time, the aforementioned strains of *C. thermoaceticum* (*M. thermoacetica*) were not known to utilize H_2_/CO_2_ and CO. Furthermore, Wiegel et al. (1981) described differences in the cell shape in comparison to *M. thermoacetica.* In addition to *C. aceticum* and *Acetobacterium woodii*, this new strain was the third species known to grow autotrophically using H_2_ and CO_2_ while producing acetate. Therefore, a new species was proposed and a strain isolated from a Yellowstone hot spring (strain JW 701/3) was deposited as *Clostridium thermoautotrophicum* DSM 1974^T^ (Wiegel et al., 1981). *C. thermoautotrophicum* was later re-classified as *Moorella thermoautotrophica* in the extensive study of Collins et al. (1994). In addition to *M. thermoautotrophica* DSM 1974^T^, which is the designated type strain, a second *M. thermoautotrophica* strain, DSM 7417, is available. This strain (DSM 7417) was first described in Rijssel et al. (1992) when it appeared as a contamination in a continuous culture. The authors based their decision to place the newly described strain in the species of *M. thermoautotrophica* instead of *M. thermoacetica* mainly on observations regarding the cell shape (Rijssel et al., 1992). Recently, Kimura et al. (2016) requested an opinion regarding the taxonomic status of *M. thermoautotrophica*. Based on DNA–DNA hybridization experiments and 16S rRNA gene sequence analysis, Kimura et al. (2016) concluded that the species *M. thermoautotrophica* should be reclassified as *M. thermoacetica*. Over time, phenotypic differences between *M. thermoacetica* and *M. thermoautotrophica* were described, but often with partly conflicting results (Cato et al., 1986; Das et al., 1989; Yamamoto et al., 1998; Carlier and Bedora-Faure, 2006).

Here, we report that *M. thermoautotrophica* DSM 1974^T^ is a mixed culture of at least two strains, which we isolated. We sequenced the genome of those two strains as well as the genome of DSM 7417 and nine other *M. thermoacetica* strains, thereby considerably adding to the genomic information of this group of bacteria. We compared the genomes of the strains with the genome of the *M. thermoacetica* strain ATCC 39073 (Pierce et al., 2008) and the type strains DSM 2955^T^ (Bengelsdorf et al., 2015) and DSM 521^T^ (Poehlein et al., 2015). In addition, we performed genome comparison with all other genomes of the genus *Moorella*. Furthermore, differences in carbon utilization of the aforementioned strains were characterized. Based on this study, we conclude that the classification of the two strains isolated from DSM 1974^T^ as a separate species, *M. thermoautotrophica*, is not justified and that based on the data collected both strains should be reclassified as strains of the species *M. thermoacetica*. However, a problem arises due to the fact that the designated type strain deposited in the DSMZ, as DSM 1974^T^, appears to be a mixture of two strains. The implications of these findings within the context of the rules of the International Code of Nomenclature (Parker et al., 2019) together with the content of the recent Request for an Opinion of Kimura et al. (2016) are discussed.

## Materials and Methods

### Strains

The strains DSM 521^T^, DSM 2955^T^, DSM 7417, DSM 21394, DSM 11768, DSM 12797, DSM 12993, DSM 6867, and DSM 11254^T^ were purchased from DSMZ (Leibniz-Institut DSMZ-Deutsche Sammlung von Mikroorganismen und Zellkulturen GmbH, Brunswick, Germany). The strains isolated from the culture of DSM 1974^T^ obtained from the DSMZ were deposited at the DSMZ with the numbers DSM 103284 (DSM 1974-Ulm) and DSM 103132 (DSM 1974-HH). Strain ATCC 39073 was purchased from the ATCC (Manassas, VA, United States) and was maintained by a series of transfers (here labeled as ATCC 39073-HH). Prior to extracting DNA for genome sequencing, a single colony was isolated on solid medium.

### Cultivation

Strains were cultivated in 50-ml serum bottles (50% filled) closed with butyl rubber stoppers (bottles and stoppers: Ochs, Germany) containing a magnetic stirring bar and medium with the following composition (in g/l) [13]: KH_2_PO_4_ (0.5); NH_4_Cl (0.4); NaCl (0.4); NaHCO_3_ (3.5); yeast extract (0.5); 1% trace element solution was added to the medium. The trace element solution was prepared with 2 g/l nitrilotriacetic acid; the pH adjusted to 6.0 with KOH, and the following compounds added (in mg/l): MnSO_4_⋅H_2_O (1000); Fe(SO_4_)_2_(NH_4_)_2_⋅6 H_2_O (800); CoCl_2_⋅6 H_2_O (200); ZnSO_4_⋅7 H_2_O (200); CuCl_2_⋅2 H_2_O (20); NiCl_2_⋅6 H_2_O (20); Na_2_MoO_4_⋅2 H_2_O (20); Na_2_SeO_4_ (20); Na_2_WO_4_ (20) mg. The pH of the culture medium was adjusted to 6.5, flushed with N_2_:CO_2_ (80:20) and autoclaved at 140°C for 40 min. Solid medium contained 1% Gelzan^TM^ and the medium was sterilized at 120°C for 20 min. The following sterile stock solutions were added after autoclaving: CaCl_2_ (50 mg/l final), MgCl_2_ (330 mg/l final), vitamin solution (1%), cysteine-HCl (1 mM final). The vitamin solution contained (mg/l): biotin (2); folic acid (2); pyridoxine-HCl (10); thiamine HCl (5); riboflavin (5); nicotinic acid (5); calcium D-(+)-pantothenate (5); vitamin B_12_ (0.5); *p*-aminobenzoic acid (5); thioctic acid (5). The medium was pre-warmed before inoculation. The strains were cultivated at 60°C with stirring at 350 rpm. Fructose as carbon and energy source was added at a final concentration of 60 mM to the medium. The headspace was pressurized with N_2_:CO_2_ (80:20) to 3 bar. When gases served as carbon and energy sources, the headspace was flushed for several minutes before inoculation with the gas mixture, and the headspace pressurized to 3 bar after inoculation. H_2_:CO_2_ (80:20) served as gaseous substrates. Strain DSM 103132 was isolated from DSM 1974^T^ using the medium described above solidified with 1% Gelzan^TM^ and using 60 mM fructose as the substrate. Strain DSM 103284 was isolated from DSM 1974^T^ using the DSMZ medium 135, the solid medium contained 1.5% agar. In both cases, single colonies were picked and used for further cultivation.

### Extraction of Genomic DNA

Cultures in mid-exponential phase were sampled, the cells were spun down, and DNA was extracted using the Wizard^®^ Genomic DNA Purification Kit (Promega, Madison, WI, United States) and the MasterPure^TM^ Gram Positive DNA Purification Kit (Epicentre, Madison, WI, United States) according to the manufacturer’s protocol. DNA was quantified using the Qubit dsDNA HS Assay Kit with the Qubit 2.0 fluorometer (Thermo Fisher Scientific, Waltham, MA, United States).

### Genome Sequencing

ATCC 39073-HH and DSM 103132 were sequenced using a PacBio RSII instrument (Pacific Biosciences, Menlo Park, CA, United States). SMRTbells^TM^ libraries were constructed and sequenced following the recommended Pacific Biosciences template preparation protocol. Following SMRTbell^TM^ construction, v2 primers and P4 polymerase were annealed and enzyme bound complexes attached to magnetic beads for loading. Each SMRTbell^TM^ library was loaded onto a SMRT cell and sequenced on the PacBio RSII. The average reference coverage was above 500 for both strains, resulting from 129,760 and 134,994 reads of ATCC 39073-HH and DSM 103132, respectively, with an average read length of approximately 12,000 bp. Isolated DNA from all remaining strains was used to generate Illumina shotgun sequencing libraries. Sequencing was performed by employing a MiSeq system using MiSeq Reagent Kit v3 (600 cycles), as recommended by the manufacturer (Illumina, San Diego, CA, United States), resulting in 2 × 300 bp paired end reads. Strain DSM 103284 was sequenced with the Genome Analyzer IIx (Illumina, San Diego, CA, United States) resulting in 2 × 112 bp paired end reads. Quality filtering of the raw reads was done using Trimmomatic version 0.32 (Bolger et al., 2014). The *de novo* assembly was performed with the SPAdes genome assembler software (Bankevich et al., 2012). The assembly was validated and the read coverage determined with QualiMap (García-Alcalde et al., 2012). For scaffolding the contigs of strain DSM 103284, we used the Move Contigs tool of the Mauve Genome Alignment Software (Darling et al., 2010). Additionally, contigs that could not be ordered with Mauve were examined via Gene Ortholog Neighborhoods based on bidirectional best hits implemented at the IMG-ER (Integrated Microbial Genomes-Expert Review) system (Markowitz et al., 2013). For contig ordering, the genomes of *M. thermoacetica* DSM 521^T^ (CP012369) and DSM 2955^T^ (CP012370) were used as references. Sequence gaps were closed by PCR-based techniques and primer walking with conventional Sanger sequencing, using BigDye 3.0 chemistry on an ABI3730XL capillary sequencer (Applied Biosystems, Life Technologies GmbH, Darmstadt, Germany), and employing the Gap4 (v.4.11) software of the Staden Package (Staden et al., 1999). *M. glycerini* DSM 11254^T^ has been sequenced using a combined approach with Illumina short read and Oxford Nanopore long read technology. Therefore, high molecular weight DNA (HWD) was isolated with the MasterPure Complete DNA & RNA Purification Kit (Biozym, Hessisch Oldendorf, Germany) as recommended by the manufacturer. Quality of isolated DNA was initially checked by agarose gel electrophoresis and validated on an Agilent Bioanalyzer 2100 using an Agilent DNA 12000 Kit as recommended by the manufacturer (Agilent Technologies, Waldbronn, Germany). Concentration and purity of the isolated DNA was first checked with a Nanodrop ND-1000 (PeqLab Erlangen, Germany), and exact concentration was determined using the Qubit^®^ dsDNA HS Assay Kit as recommended by the manufacturer (Life Technologies GmbH, Darmstadt, Germany). Illumina shotgun libraries were prepared using the Nextera XT DNA Sample Preparation Kit and subsequently sequenced on a MiSeq system with the reagent kit v3 with 600 cycles (Illumina, San Diego, CA, United States) as recommended by the manufacturer resulting in 1,694,377 paired end reads. For Nanopore sequencing, 1.5 μg HWD was used for library preparation using the Ligation Sequencing Kit 1D (SQK-LSK109) and the Native Barcode Expansion Kit (EXP-NBD104) as recommended by the manufacturer. Sequencing was performed on a MinION device Mk1B using a SpotON Flow Cell R9.4.1 as recommended by the manufacturer for 72 h. This resulted in 162,721 reads with a mean read length of 4,155 bp. Unicycler v0.4.8 (Wick et al., 2017) was used with default settings to perform a hybrid assembly.

### Genome Annotation

The genomes were annotated using the Prokka automatic annotation software (Seemann, 2014). Protein coding, rRNA, and tRNA sequences were annotated using Prodigal (Hyatt et al., 2010), RNAmmer (Lagesen et al., 2007), and Aragorn (Laslett and Canback, 2004) against databases using BLAST (Camacho et al., 2009) and HMMER (Finn et al., 2011). Prediction of non-coding RNAs and CRISPR repeats were done by infernal (Nawrocki and Eddy, 2013) and MinCED^1^ based on CRISPR Recognition Tool (Bland et al., 2007). Signal peptides were searched using SignalP (Petersen et al., 2011). Protein coding genes were analyzed for COG (Tatusov et al., 1997; Galperin et al., 2014) functional annotation using Batch CD-search tool (Marchler-Bauer and Bryant, 2004). No secondary metabolite clusters were predicted in an analysis using antiSMASH 2.0 (Medema et al., 2011). The genome sequences and annotation data of all *M. thermoacetica* strains have been deposited in DDBJ/ENA/GenBank, for detailed information see Table 1.

**TABLE 1:** Genome features of *Moorella* species.

**Organism**	**Accession number**	**Size [bp]**	**GC-content [%]**	**Coding percentage [%]**	**CDS**	**Genes**	**rRNA**	**tRNA**	**Contigs**	**References**
*M. glycerini* DSM 11254^T^	CP046244 (chromosome) CP046245 (plasmid)	3,559,463	54,74	88.26	3,509	3,564	3	52	2	This study
*M. glycerini* NMP	CELZ01000000	3,577,805	53.80	88.48	3,636	3,697	4	57	73	Liebensteiner et al., 2015
*M. humiferrea* DSM 23265^T^	PVXM00000000	2,628,568	53.52	89.51	2,668	2721	3	49	63	Poehlein et al., 2018b
*M. mulderi* DSM 14980^T^	LTBC00000000	3,307,499	54.54	87.26	3,042	3,099	3	53	72	Castillo Villamizar and Poehlein, 2016
*M. perchloratireducens* An10	Gp0011525^a^	3,307,499	53.84	88.69	3,349	3,423	3	52	133	Markowitz et al., 2013
*M. stamsii* DSM 26217^T^	PVXL00000000	3,328,173	53.81	87.87	3,306	3,358	3	49	82	Poehlein et al., 2018a
*M. thermoacetica* ATCC 31490	VCDV00000000	2,616,798	55.81	87.94	2,621	2,676	3	51	26	This study
*M. thermoacetica* ATCC 33924^b^	VCDY00000000	2,914,842	55.12	87.81	2,959	3,020	3	57	47	This study
*M. thermoacetica* ATCC 35608	VCDW00000000	2,611,625	55.83	87.96	2,605	2,661	3	52	26	This study
*M. thermoacetica* ATCC 39073	CP000232	2,628,784	55.79	86.39	2,465	2,634	3	51	1	Pierce et al., 2008
*M. thermoacetica* ATCC 49707	VCDX00000000	2,616,845	55.83	87.92	2,619	2,676	4	52	28	This study
*M. thermoacetica* DSM 103132	CP017019	2,976,077	55.10^c^	87.94	3,026	3,091	6	58	1	This study
*M. thermoacetica* DSM 103284	CP017237	2,560,375	55.94^c^	87.94	2,525	2,58	3	51	1	This study
*M. thermoacetica* DSM 11768	MIHH00000000	2,851,436	55.66	86.40	2,806	2,865	3	55	92	This study
*M. thermoacetica* DSM 12797	MIIF00000000	2,746,010	55.54	86.51	2,716	2,774	3	54	83	This study
*M. thermoacetica* DSM 12993	MDDD00000000	2,648,948	55.74	87.10	2,6	2,659	3	55	40	This study
*M. thermoacetica* DSM 21394	MDDC00000000	2,567,468	56.95	85.80	2,499	2,559	3	56	30	This study
*M. thermoacetica* DSM 2955^T^	CP012370	2,623,349	55.81	88.17	2,624	2,68	3	52	1	Bengelsdorf et al., 2015
*M. thermoacetica* DSM 512^T^	CP012369	2,527,564	55.95^d^	88.06	2,553	2,609	3	52	1	Poehlein et al., 2015
*M. thermoacetica* DSM 6867	MDDB00000000	2,617,097	55.83	87.80	2,62	2,676	3	52	42	This study
*M. thermoautotrophica* DSM 7417	MDDE00000000	2,585,122	55.87	87.59	2,558	2,62	6	55	26	This study
*M. thermoacetica* Y72	BARR00000000	2,603,418	55.89	88.05	2,629	2,715	3	51	95	Tsukahara et al., 2014

*^*a*^GOLD Sequencing Project ID (no accession number available). ^*b*^DNA isolated and sequenced from a single colony. ^*c*^Wiegel et al. (1981) stated a GC content of 53–55% in the DNA of strain DSM 1974^*T*^. ^*d*^Matteuzzi et al. (1978) stated a GC content of 54% in the DNA of strain DSM 521^*T*^.*

### Genome Analysis

For MLSA and gene content analysis, total protein sequences from the 24 genomes were extracted from the corresponding GenBank files using cds_extractor.pl v0.6^2^ and used for downstream analysis with an in-house pipeline at the Göttingen Genomics Laboratory. In detail, proteinortho version 4.25 (default specification: blast = blastp v2.2.24, E-value = 1e-10, alg.-conn. = 0.1, coverage = 0.5, percent_identity = 50, adaptive_similarity = 0.95, inc_pairs = 1, inc_singles = 1, selfblast = 1, unambiguous = 0) (Lechner et al., 2011) was used to generate clusters of orthologs groups, inparalogs were removed. MUSCLE (Edgar, 2004) was employed to align the remaining sequences and poorly aligned positions were automatically filtered from the alignments using Gblocks (Castresana, 2000). A maximum likelihood tree from 1,177 orthologs groups was inferred with 500 bootstraps with RAxML (Stamatakis, 2014). The script PO_2_MLSA.py is available at github^3^. Visualization of the tree was performed using Dendroscop (Huson and Scornavacca, 2012).

Average Nucleotide Identity (ANIm) analyses were performed using pyani.py^4^. Briefly, nucleotide sequences were extracted from the corresponding GenBank files using seq_format-converter.pl v0.2^5^ and subsequently used to run pyani in ANIm mode (uses MUMmer/NUCmer) to align input sequences. PHASTER (PHAge Search Tool Enhanced Release, Arndt et al., 2016) has been used for the detection of prophage regions. The analysis of genomic islands was performed using IslandViewer 4 (Bertelli et al., 2017).

## Results

Strain DSM 1974^T^ was purchased from DSMZ by our labs (University of Ulm and Technical University of Denmark) separately in 2015. Genome sequencing of the strain in the Göttingen Genomics Laboratory and at the Technical University of Denmark suggested that DSM 1974^T^ is a mixed culture. After suspecting cross-contamination in our labs, new DSM 1974^T^ cultures were ordered from DSMZ, however, with the same result. We independently isolated single clones after cultivation of DSM 1974^T^ on solid medium: DSM 103284 (DSM 1974-Ulm) at the University of Ulm and DSM 103132 (DSM 1974-HH) at the Technical University of Denmark as described in Section “Materials and Methods.” We sequenced the genome of both strains which were derived from the DSM 1974^T^ culture, as well as the genome of DSM 7417 and the genome of another ATCC 39073 strain, here designated ATCC 39073-HH. In order to determine whether DSM 1947^T^ is a mixed culture we ordered ATCC 33924^T^ (that is derived from DSM 1974^T^) from the ATCC and sequenced the DNA directly isolated from the freeze-dried culture (data not shown) and from a single colony isolated with the same procedure as for strain DSM 103132. Sequencing results confirmed that ATCC 33924^T^ = DSM 1974^T^ deposited at the ATCC is also a mixed culture and the strain isolated from that culture is identical to DSM 103132. The differences between DSM 103132 (isolated in Denmark) and DSM 103284 (isolated in Germany) suggest that slightly different cultivation conditions may favor the selection of different strains from the original mixed culture of DSM 1974^T^. In addition, the genomes of 10 different *M. thermoacetica* strains, *M. thermoautotrophica* DSM 7417, and *M. glycerini* DSM 11254^T^ were sequenced.

### Genome Features

Table 1 shows an overview of the *de novo* sequenced genomes of the DSM 1974^T^-derived strains (DSM 103284 and DSM 103132) and all other strain sequences in this study compared to the published genomes of type strains DSM 521^T^, DSM 2955^T^, as well as ATCC 39073, *M. thermoacetica* Y72, *M. glycerini* DSM 11254^T^, *M. glycerini* NMP, *M. humiferrea* DSM 23268^T^, *M. mulderi* DSM 14980^T^, *M. perchloratireducens* An10, and *M. stamsii* DSM 26217^T^. In order to investigate the phylogeny of the strains, we first compared the 16S rRNA gene sequences of the type strains, ATCC 39073-HH, DSM 103132, and DSM 103284. The sequence similarity between the strains in the 16S rRNA gene region is at least 99.74%, as no more than 3 nucleotide mismatches could be found. In strains DSM 103284 and ATCC 39073-HH, the gene regions are identical. According to Stackebrandt and Goebel (1994), bacteria showing less than 97% similarity in their 16S rRNA gene sequences belong to different species, while additional methods must be taken into consideration when the 16S rRNA similarity values are above 97%. All strains were analyzed with respect to prophages and interestingly none of the strains harbors a complete prophage. In all strains, a different number of incomplete phages (between 1 and 5; for details see Supplementary Table S2) were detected. Six strains, *M. thermoacetica* DSM 103132 and DSM 103284, *M. glycerini* DSM 11254^T^ and NMP, *M. perchloratireducens* An10, and *M. stamsii* DSM 26217^T^ contain putative phages, marked as “questionable.” These DNA regions show similarity to different *Bacillus* phages or to a Stx2-converting phage (Supplementary Table S2). We also checked some completely sequenced strains for the presence of genomic islands and found 9 such regions in strains DSM 103284 and DSM 2955^T^ as well as 10 genomic islands in strain DSM 521^T^ and ATCC 39073. Strain DSM 103132 harbors 36 genomic islands in total and one of these regions has a size of 166 kbp (Supplementary Table S3). All genomic islands contain mainly hypothetical proteins, transposases, or transcriptional regulators and only a few genes coding for enzymes (for details see Supplementary Table S3). With respect to plasmids, a 50-kbp plasmid was found in *M. glycerini* DSM 11254^T^. None of the *M. thermoacetica* or *M. thermoautotrophica* strains was found to carry a plasmid. All other *Moorella* species could not be analyzed in detail, as they are draft genomes and there is no evidence for plasmid replication genes in these genomes.

### Phylogenetic Analysis

**FIGURE 1:**
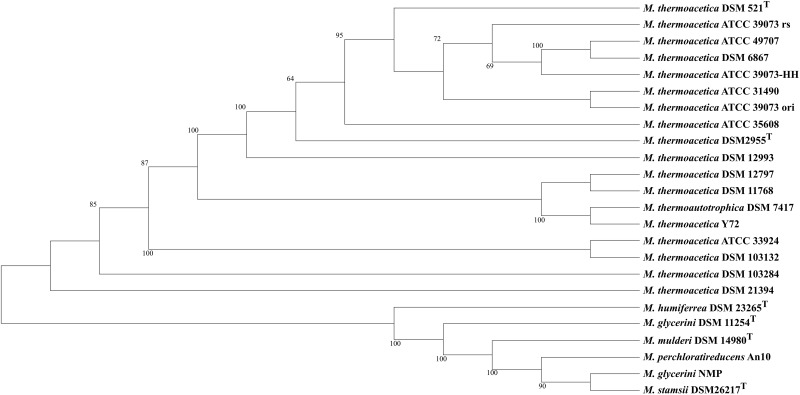
MLSA tree of 24 sequenced *Moorella* strains: maximum likelihood trees of 24 *Moorella* genome sequences were inferred with 500 repetitions with RAxML (Stamatakis, 2014) and visualized with Dendroscope (Huson and Scornavacca, 2012). *M. thermoactica* marked with ATCC 39073 ori is the original sequence of this strain, ATCC 39073 rs is a sequenced version of the genome performed by the JGI and ATCC 39073-HH is a sequenced version of the genome performed by Technical University of Denmark.

**FIGURE 2:**
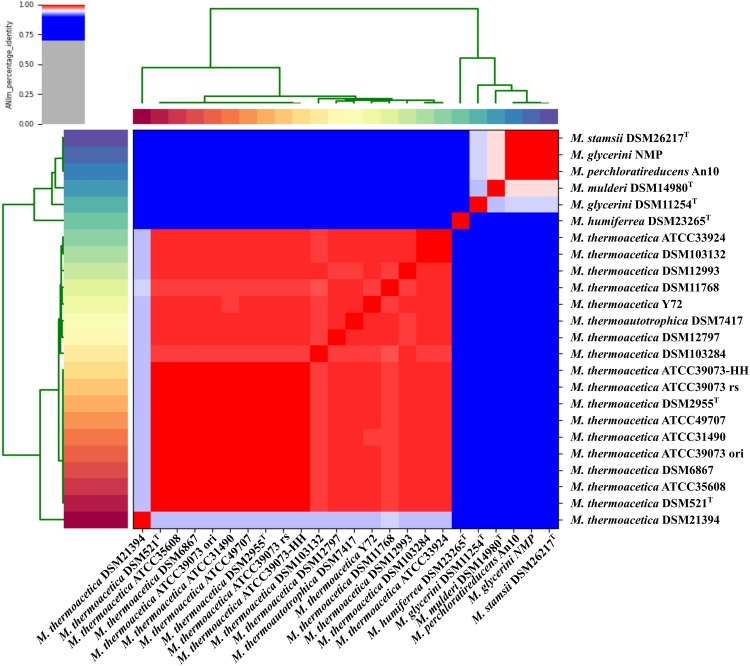
Average nucleotide identity analysis of the 24 sequenced strains: ANIm analysis based on MUMmer alignment (Delcher et al., 2002) of the genome sequences was performed and visualized using PYANI (https://github.com/widdowquinn/pyani). *M. thermoactica* marked with ATCC 39073 ori is the original sequence of this strain, ATCC 39073 rs is a sequenced version of the genome performed by the JGI and ATCC 39073-HH is a sequenced version of the genome performed by Technical University of Denmark.

We used MLSA based on the detected core genome (1,177 OGs excluding paralogs) to perform phylogenetic analysis of our strains (Figure 1) and an average nucleotide identity analysis (ANIm) (Figure 2). The phylogenetic tree yielded two main clades, one consisting of all *M. thermoacetica* and *M. thermoautotrophica* strains and the second of *M. glycerini* DSM 11254^ T^, *M. glycerini* NMP, *M. humiferrea* DSM 23268^T^, *M. mulderi* DSM 14980^T^, *M. perchloratireducens* An10, and *M. stamsii* DSM 26217^T^. The first main clade shows three distinct subclades, one consisting of the different versions of *M. thermoacetica* ATCC 39079, DSM 521^T^, DSM 2955^T^, ATCC 49707, ATCC 31490, ATCC 35608^T^, DSM 12993 and DSM 6867. It should be noted that DSM 521^T^, DSM 2955^T^, ATCC 35608^T^, and ATCC 49707 are all derived from the same original strain. The second subclade consists of strains DSM 12797, DSM 11786, Y72, and DSM 7417. Strains ATCC 33924 and DSM 103132 form the third subclade. Strain DSM 103284, isolated from the mixed culture of DSM 1974^T^ and strain DSM 21394 cluster outside of the three subclades. Interestingly, DSM 21394 is the strain with the third highest number of singletons (300 OGs). Whilst MLSA can provide insight into the phylogenetic relationship of organisms, for taxonomic studies there is a requirement for other methods, such as ANI analysis (Richter and Rosselló-Móra, 2009), which is a suitable *in silico* alternative for DNA–DNA hybridization (Goris et al., 2007). We performed an ANI analysis based on MUMmer alignment (ANIm) of the 24 genomes to define species and their complexes (Figure 2). We identified a large cluster comprising all *M. thermoacetica* and *M. thermoautotrophica* strains including DSM 103132 and DSM 103284, which have been both re-isolated from DSM 1974^T^ as well as DSM 7417. The latter two strains are currently considered to be *M. thermoautotrophica* strains. However, our analysis clearly shows that these strains would be more appropriately classified as *M. thermoacetica* isolates, since we identified ANIm values between 98 and 99% compared to *M. thermoacetica* DSM 512^T^ and DSM 2955^T^, the two independent deposits of the type strain of this species in the DSMZ (Supplementary Table S1). Richter and Rosselló-Móra (2009) proposed a threshold for the species boundary of 95% ANI, making reference to both ANIb and ANIm values. However, careful examination of their original data suggests that ANIb and ANIm do not give the same values and the species boundary for the two may be different. ANIm values of 98–99% are clearly above this threshold, but values of 95–96% need to be taken with caution. Our analysis also revealed that strain DSM 21394 has an ANIm value of 94% (Supplementary Table S1) compared to the other *M. thermoacetica* strains, which is below the threshold for the species boundary and further studies would be needed to determine whether this strain should also be re-classified. This is also depicted in Figure 2, where all strains belonging to one species are marked in red tones. Interestingly, *M. stamsii* DSM 26217^T^, *M. glycerini* NMP, and *M. perchloratireducens* An10 showed an ANIm value of 100% and they should therefore belong to the same species. However, the name *M. perchloratireducens* has not been validly published and *M. glycerini* NMP is not the nomenclatural type of the species so no formal nomenclatural action is required under the International Code of Nomenclature of Prokaryotes (Parker et al., 2019), since the names *M. glycerini* and *M. perchloratireducens* can only be formally considered to be heterotypic synonyms if both are validly published and are the corresponding nomenclatural types. The ANIm value of the type strain of *M. stamsii* to the other two strains is also 100%, indicating that all three should be placed in the same species, i.e., *M. stamsii*, which has been validly published. These results are in contrast to the published viewpoint that *M. stamsii* and *M. perchloratireducens* represent distinct species. It is common practice to determine the 16S rRNA gene sequence of a novel isolate and initially investigate the similarity value to the 16S rRNA gene sequences of other type strains before deciding how to further characterize a strain. In the case of 16S rRNA gene sequence similarity values of 97% and greater it is common practice to determine DNA–DNA hybridization values (which is now being replaced by ANI or digital DNA–DNA hybridization studies) to evaluate whether one is dealing with a new species. Where the 16S rRNA gene sequence similarity values are less than 97%, it is generally assumed that one has a novel species. Key discrepancies in the study of *M. glycerini, M. stamsii*, and *M. perchloratireducens* are the 16S rRNA gene sequences and the genomic similarity. In the case of *M. glycerini*, the 16S rRNA gene sequence determined in the original study (Slobodkin et al., 1997), U82327, showed a pairwise similarity of 99.3% to the 16S rRNA sequence determined in the genome (CP046244). The 16S rRNA gene sequence determined in the original study of *M. stamsii* (Alves et al., 2013), HF563589, showed a pairwise similarity of 99.3% to the 16S rRNA sequence determined in the genome contig PVXL01000051. When U82327 and HF563589 were compared by Alves et al. (2013), the similarity values were 97%, but comparison of the 16S rRNA gene sequences obtained from the genomes (CP046244 and PVXL01000051) now gives 99.2% similarity and 100% similarity to CELZ01000013. In the case of DNA–DNA hybridization between these two strains the value was 51.1–53.3% (duplicated measurements). The 16S rRNA gene sequence from the genome of *M. glycerini* DSM 11254^T^ contains a large deletion that does not occur in U82327 or any of the other PCR-amplified 16S rRNA gene sequences or those determined via genome sequencing of the same strain (Supplementary Figure S1). The PCR-amplified 16S rRNA gene from *M. stamsii* (HF563589) also appears to contain numerous additional bases.

In the case of *M. perchloratireducens*, comparison of the 16S rRNA gene sequence determined in the original study, EF060194 (Balk et al., 2008), with that extracted from the genome (Gp0011525) showed 95.1% similarity. While EF060194 showed 97% sequence similarity with the 16S rRNA gene sequence from the genome of *M. thermoacetica* ATCC 39073 (CP00232), comparisons with the 16S rRNA gene sequence from Gp0011525 indicated that the genome-derived sequences showed 95% sequence similarity. In contrast, comparisons between EF060194 (*M. perchloratireducens*) and U82327 (*M. glycerini*)/HF563589 (*M. stamsii*) gave sequence similarity values of 93.9 and 93.1%, respectively. However, comparisons based on the 16S rRNA gene sequences extracted from the genomes Gp0011525 (*M. perchloratireducens*), CP046244 (*M. glycerini*), and PVXL01000051 (*M. stamsii*) gave pairwise similarities of 99.2–100%. No DNA–DNA hybridization studies were carried out by Balk et al. (2008), because they used a 16S rRNA gene sequence “threshold” of 98% 16S rRNA similarity. These results suggest significant discrepancies between the 16S rRNA gene sequence EF060194 obtained by primer amplified sequencing and that determined by genome sequencing that are evident in the alignments (Supplementary Figure S1) and are difficult to attribute to experimental error without further confirmatory work. It is interesting to note that of the two deposits of *M. perchloratireducens* An10, ATCC BAA-1531 and JCM 14829 only ATCC BAA-1531 is currently available and is the source strain for the genome Gp0011525. In the case of *M. mulderi* DSM 14980, the genome-derived 16S rRNA gene sequence (LTBC01000042.1) contains a large insert not present in sequence of the original PCR-amplified gene deposited as AF487538.1 (Supplementary Figure S1).

The ANIm values between *M. glycerini* (strain NMP), *M. stamsii*, and *M. perchloratireducens* indicate that they belong to the same species. Although the 16S rRNA gene sequence of the type strains of *M. glycerini* and *M. stamsii* are 99.3%, the AMIm value of 94% indicates that they are different species. In the case of *M. mulderi* DSM 14980 the genome-based 16S rRNA gene sequence similarity to *M. glycerini* DSM 11254 is 98.8% and the AMIm value 93%, indicating that they are different species. When compared to the genome-based 16S rRNA gene sequences of *M. glycerini* (strain NMP), *M. stamsii*, and *M. perchloratireducens* the value is 99.3% and the ANIm value 96%; this would appear to indicate that *M. mulderi* DSM 14980 is a member of the same species as *M. stamsii* DSM 26217. However, the original work of Richter and Rosselló-Móra (2009) indicate that an ANI cut-off of 95% ANIb is equivalent to an ANIm value of 96.5%, indicating that *M. mulderi* DSM 14980 and *M. stamsii* DSM 26217 are not members of the same species. This work also indicates the importance of examining the data beyond simple similarity values, where examination of the individual 16S rRNA gene sequence alignments, the differences in gene content and genome size provide extra valuable detail.

### Genome Comparison

**FIGURE 3:**
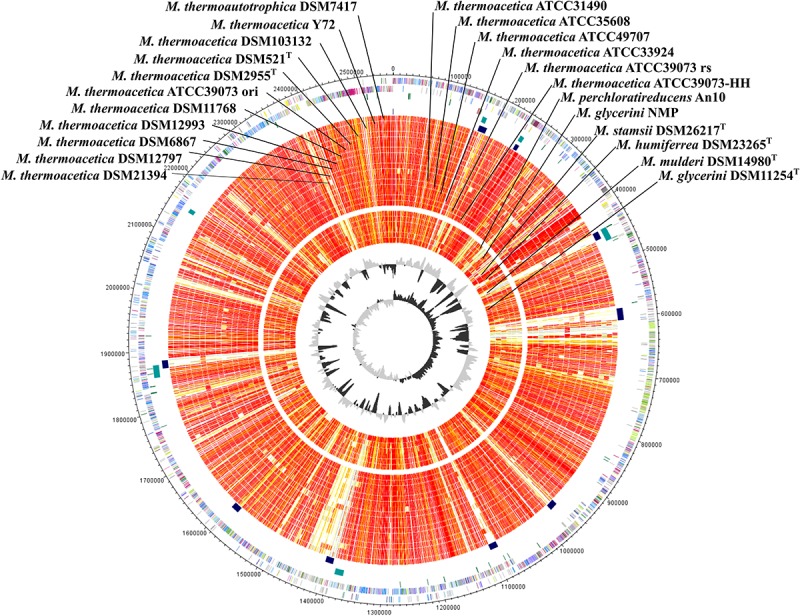
Circular representation of the genome comparison of *M. thermoacetica* DSM 103284 with other *Moorella* strains. The genes encoded by the leading and the lagging strand (outer circles 1 and 2) of *M. thermoacetica* DSM 103284 are marked in COG colors in the artificial chromosome map. tRNA (green) and rRNA (pink) genes were plotted on circle 3. Detected prophage regions (petrol) and genomic islands (dark blue) are shown on circles 4 and 5, respectively. The presence of orthologous genes (red, high similarity; orange, medium similarity; yellow, low similarity (see color code below) is indicated for the genomes in comparison to *M. thermoacetica* DSM 103284. The two innermost plots represent the GC content and the GC skew (circles 29 and 30). Visualization was done using Proteinortho (Lechner et al., 2011) results and DNAPlotter (Carver et al., 2009). COG categories of the genes were extracted from IMG database (Galperin et al., 2014) entries of *M. thermoacetica* DSM 103284. Color code according to E-values of the blastp analysis performed using Proteinortho4.26. Gray, 1e^–20^ to 1; light yellow, 1e^–21^ to 1e^–50^; gold, 1e^–51^ to 1e^–90^; light orange, 1e^–91^ to 1e-^100^; orange, 1e^–101^ to 1e^–120^; red, > 1e^–120^ *M. thermoactica* marked with ATCC 39073 ori is the original sequence of this strain, ATCC 39073 rs is a sequenced version of the genome performed by the JGI and ATCC 39073-HH is a sequenced version of the genome performed by Technical University of Denmark.

**FIGURE 4:**
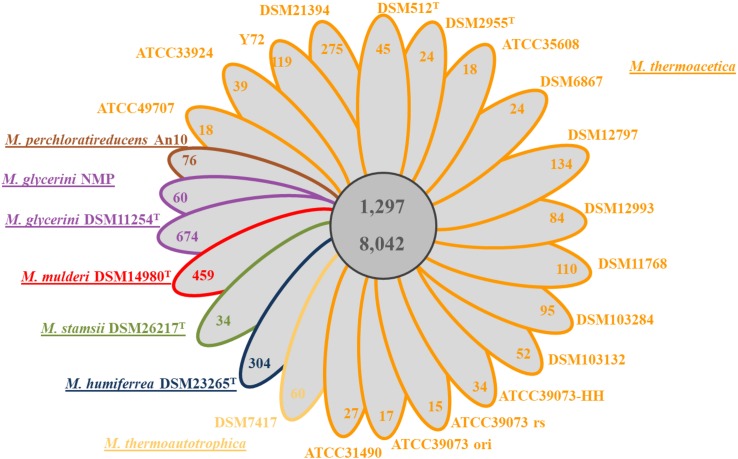
Core/Pan genome analysis of 24 *Moorella* genomes: a simplified Venn diagram showing the core and the pan genome of all 24 *Moorella* strains. The number of genome-specific OGs is depicted in the respective ellipse. Ortholog detection was done with blastp and the Proteinortho software (Lechner et al., 2011) with a similarity cut off of 50% and an E-value of 1e^–10^. *M. thermoacetica* marked with ATCC 39073 ori is the original sequence of this strain, ATCC 39073 rs is a sequenced version of the genome performed by the JGI and ATCC 39073-HH is a sequenced version of the genome performed by the Technical University of Denmark.

Until recently, only the sequence of the non-type strains *M. thermoacetica* ATCC 39079 and *M*. *thermoacetica* Y72 were publicly available, but many other strains, including the two independently deposited type strains of the species (DSM 521^T^ and DSM 2955^T^), and several other strains are available at the German Collection of Microorganisms and Cell Cultures (DSMZ Brunswick), including strain DSM 1974^T^. We sequenced the genomes of all these strains and performed whole genome comparison of all *M. thermoacetica* strains, and comparison with the genomes of five other species, namely *M. stamsii* DSM 26217^T^, *M. humiferrea* DSM 23265^T^, *M. glycerini* DSM 11254^T^, M. *glycerini* NMP, *M. perchloratireducens* An10, and *M. mulderi* DSM 14980^T^ (Figure 3). All *M. thermoacetica* strains have a comparable genome size of 2.52–2.64 Mb, except the two closely clustering strains DSM 103132 and ATCC 33924, which have larger genomes (2.98 and 2.91 Mb). *M. glycerini* NMP has the largest genome size in our comparison with 3.58 Mb, followed by *M. glycerini* DSM 11254^T^ with 3.56 Mb. A whole genome comparison based on protein encoding genes revealed a core genome shared by all 24 strains of 1,297 OGs including paralogs and a pan genome of 8,042 OGs (Figure 4). The pan genome includes the core and the flexible genome, OGs shared by at least two genomes, but not by all genomes in the comparison. The size of the core genomes is half the size of the complete genome of the *M. thermoacetica* strains, due to the high proportion of *M. thermoacetica* strains in our comparison. We found a broad range of singletons, meaning genome-specific genes, varying between 15 and 275 OGs in the *M. thermoacetica* group. The highest number of singletons (674 OGs) was found in the genome of *M. glycerini* DSM 11254^T^. The flexible genome harbors for example a complete gene cluster encoding a pyruvate:ferredoxin oxidoreductase, which is only present in DSM 103284, DSM 11768, DSM 512^T^, DSM 2955^T^, DSM 12797, and all ATCC 39073 genomes. A cluster encoding an anaerobic dimethylsulfoxide reductase (DSMO reductase) is present in all genomes compared here, except of *M. mulderi* DSM 14980 and *M. thermoacetica* DSM 103132, which has been re-isolated from the mixed culture DSM 1974. We also identified OGs that are specific for the above-mentioned phylogenetic clades. We identified, for example, a gene cluster coding for a carbohydrate-specific ABC transport system, which is exclusively present in the first main clade comprising all *M. thermoacetica* strains, but which is absent in the second main clade consisting of *M. stamsii* DSM 26217^T^, *M. humiferrea* DSM 23265^T^, *M. glycerini* DSM 11254^T^, *M. perchloratireducens* An10, and *M. mulderi* DSM 14980^T^. We also identified gene clusters specific for the first main clade, for example a cluster encoding, amongst other genes, a ribose permease, L-rhamnose mutarotase, and a L-fucose isomerase probably involved in rhamnose and fucose metabolism. There are also genome-specific genes. *M. thermoacetica* DSM 103284 for example harbors a hydrogenase gene cluster that could not be identified in any other genome analyzed in this study.

### Phenotypical and Physiological Differences Between *M. thermoacetica* and *M. thermoautotrophica* Strains

Several phenotypical and physiological differences between *M. thermoacetica* and *M. thermoautotrophica* strains regarding compounds that can serve as carbon and energy source have been described in the literature. Those results are sometimes contradictory to each other. We therefore tested whether there are differences between the strains regarding carbon source utilization and whether the results can give a hint toward the identity of strain DSM 1974^T^. Our results largely agree with the results reported in the literature. Within the tested strains, only DSM 103132 can utilize arabinose (Table 2). As already published, *M. thermoacetica* (DSM 521^T^ or ATCC 39073) (Fontaine et al., 1942; Andreesen et al., 1973; Cato et al., 1986) and DSM 1974^T^ (Wiegel et al., 1981; Cato et al., 1986) are not able to utilize arabinose. DSM 103132 was also found to be the only strain that could utilize formate, but only reaching low optical densities. DSM 1974^T^ has been reported to utilize formate (Wiegel et al., 1981; Fröstl et al., 1996), like ATCC 39073 (Fröstl et al., 1996). All tested strains were able to grow on fructose and glucose, and these substrates led to the highest cell density, which is in agreement with literature on *M. thermoacetica* (DSM 521^T^ or ATCC 39073) (Fontaine et al., 1942; Andreesen et al., 1973). DSM 521^T^ was the only strain that did not utilize H_2_ + CO_2_ as carbon and energy source in our experiments. ATCC 39073 (Daniel et al., 1990; Fröstl et al., 1996) and DSM 1974^T^ (Wiegel et al., 1981; Savage and Drake, 1986; Fröstl et al., 1996) have been reported to utilize methanol. In agreement with our results, DSM 521^T^ has been reported not to grow on methanol (Cato et al., 1986). All strains tested in this study grew with pyruvate as energy and carbon source. Interestingly, Cato et al. (1986) indicated that pyruvate does not serve as a growth-supportive substrate for DSM 1974^T^ (Wiegel et al., 1981). None of the tested strains, except for DSM 103284, could utilize rhamnose, which is in line with DSM 1974^T^ being the only *M*. *thermoacetica/thermoautotrophica* strain previously reported to utilize rhamnose (Cato et al., 1986). According to the literature, *M. thermoacetica* (DSM 521^T^ or ATCC 39073) (Fontaine et al., 1942; Andreesen et al., 1973; Cato et al., 1986) and DSM 1974^T^ (Wiegel et al., 1981; Cato et al., 1986) are not capable of utilizing sucrose, however, we observed growth for ATCC 39073-HH and DSM 103132 on that substrate. All tested strains, except DSM 103132, utilized xylose. In the case of DSM 1974^T^, contradictory results have been reported: according to Cato et al. (1986), in 61–89% of the tests, cultures were able to utilize xylose. Some of the differences in substrate utilization can be explained by comparison of the genomes. For example, the arabinose operon in the genome of DSM 103132 is not present in the genome of ATCC 39073-HH. The pathway for xylose utilization is encoded in the ATCC 39073-HH genome, but not in the DSM 103132 genome. Other differences in carbon source utilization between the various studies may be due to the fact that strains might have adapted to different substrates or that the substrate utilization depends on the growth stage of the inoculum (Wiegel et al., 1981), which may be caused by differences in transcriptional regulators between the strains (Marcellin et al., 2016). Our results do not allow an unambiguous conclusion to be drawn whether one of the strains (DSM 103132 and DSM 103284) corresponds to the strain originally studied by Wiegel et al. (1981) and deposited as DSM 1974^T^ in the DSMZ. In addition to carbon source utilization, other phenotypical and physiological differences between the *M. thermoacetica/thermoautotrophica* strains have been described, such as differences in motility [DSM 1974^T^ is motile (Cato et al., 1986), DSM 521^T^ is not (Carlier and Bedora-Faure, 2006)] and growth temperature [DSM 1974^T^ can grow at 70°C (Cato et al., 1986), while DSM 521^T^ cannot (Carlier and Bedora-Faure, 2006)].

**TABLE 2:** Substrate utilization by selected *Moorella* strains.

		**ATCC 39073-HH**	**DSM 103132**	**DSM 2955^T^**	**DSM 521^T^**	**DSM 103284**	**DSM 7417**	**Data for original strain DSM 1974^T^ (Wiegel et al., 1981)**
Arabinose	60 mM	NG	0.42	NG	NG	NG	NG	NG
Formate	10 mM	NG	0.12	NG	NG	NG	NG	+
Fructose	60 mM	1.65	1.30	1.66	0.67	1.24	1.29	+ +
Glucose	60 mM	1.50	0.22	1.90	0.59	0.25	0.23	+ +
H_2_/CO_2_	30 psi	0.11	0.22	0.45	NG	0.28	0.36	+
Methanol	60 mM	0.22	0.10	0.49	NG	0.50	0.26	+
Pyruvate	60 mM	0.39	0.33	0.28	0.33	0.56	0.27	NG
Rhamnose	60 mM	NG	NG	NG	NG	0.60	NG	NR
Sucrose	60 mM	1.60	0.26	NG	NG	NG	NG	NG
Xylose	60 mM	1.50	NG	0.88	0.44	0.70	0.70	NG

*The highest optical densities are reported that were measured for the respective carbon sources. NG, no growth; NR, not reported; +, slow growth or low optical density; + +, fast growth, optical density at 600 nm above 1.0 with 0.5% (wt/vol) carbon source after 4 days.*

## Discussion

Strains of *M. thermoacetica* and *M. thermoautotrophica* have become model organisms of the acetogenic metabolism. Due to the observation of conflicting phenotypic traits that have been connected with the two different species, the scientific community has already questioned the taxonomic status of the two species *M. thermoautotrophica* and *M. thermoacetica* (Carlier and Bedora-Faure, 2006; Kimura et al., 2016). In addition to the high similarity of the genomes’ 16S rRNA gene sequence, there are further similarities described for *M. thermoacetica/thermoautotrophica* strains such as a similar fatty acid and peptidoglycan profile (Yamamoto et al., 1998) and presence of the same menaquinone (Das et al., 1989). However, these features are generally conserved in “closely related” taxa and one would not expect significant differences between strains showing such a high degree of genetic similarity (Tindall, unpublished). Until a few years ago, only the sequence of the non-type strains *M. thermoacetica* ATCC 39079 and *M. thermoacetica* Y72 were publicly available, but many other strains, including the two type strains of the species (DSM 521^T^ and DSM 2955^T^), and several other strains are available at the German Collection of Microorganisms and Cell Cultures (DSMZ Brunswick), including strain DSM 1974^T^. We wished to broaden knowledge of the genetic diversity of this group of organisms and therefore sequenced the genome of both strains which were derived from the DSM 1974^T^ culture (DSM 103132 and DSM 103284), as well as the genome of DSM 7417 and the genome of another sub-culture of ATCC 39073 (ATCC 39073-HH). In addition, the genomes of eight different *M. thermoacetica* strains were sequenced. Comparison of the 16S rRNA gene sequences of the strains, ATCC 39073(-HH), DSM 103132, and DSM 103284, showed a sequence similarity between the strains higher than 99.74%. We used MLSA, gene content analysis, and ANI analysis to get insights into the phylogeny of the genus *Moorella*. With ANIm values between 98 and 99% compared to the other *M. thermoacetica* strains DSM 512^T^ and DSM 2955^T^, the strains derived from DSM 1974^T^ (DSM 103132 and DSM 103284) are clearly *M. thermoacetica* isolates. Through genome sequencing of different *M. thermoacetica* and *M. thermoautotrophica* strains, it was evident that *M. thermoautotrophica* DSM 1974^T^ consists of at least two different strains, which are both very closely related to each other and to *M. thermoacetica*. Since phylogenetic analysis showed that all *M. thermoacetica/thermoautotrophica* strains described to date belong to the same species, there would appear to be no justification based on the currently available data for considering *M. thermoautotrophica* to be a separate species. Consequently, the strains DSM 103132 and DSM 103284 (both derived from DSM 1974^T^, the designated type strain of *M. thermoautotrophica*) must be designated as *M. thermoacetica*. Based on the current study, the observed phenotypic differences are likely to be due to strain variations within one species, as already indicated by Wiegel et al. (1981) and Cato et al. (1986). Furthermore, observed differences in carbon source utilization cannot serve as a suitable measure to distinguish species, since the substrate acceptance may be dependent on cultivation conditions. However, the picture is complicated by the fact that DSM 1974^T^, the strain which led to the proposal of the new species *C. thermoautotrophicum* (Wiegel et al., 1981) and was later transferred to the genus *Moorella* as *M. thermoautotrophica* (Collins et al., 1994) was consistently shown by genome sequencing to consist of two different strains. The isolation of two different strains that have subsequently been deposited as DSM 103132 and DSM 103284 confirms these observations. However, taking the original data of Wiegel et al. (1981) and comparing them with the data collected in this study for DSM 103132 and DSM 103284 does not show a large number of significant differences in the physiology of the strains. Based on the current data and taking into consideration the methods originally used by Wiegel et al. (1981) it is not possible to determine whether the original strain of Wiegel, JW 701/3, was a mixture of two different strains of the same species, whether the original strain was a pure culture, but a mixed culture was submitted for deposit (that methods used at the time would not have detected), or whether a second strain was introduced into the culture subsequent to accession to the DSMZ. Cross-contamination of strains is one possible explanation: the spores of *Moorella* species are highly heat-resistant and are not sufficiently inactivated by a standard autoclaving at 121°C (Fontaine et al., 1942). Byrer et al. (2000) for example described the strains JW/DB-2 and JW/DB-4 (ATCC number BAA-48) that show unusually heat-resistant spores. However, given the resolution of methods used at the time, one also cannot exclude with certainty that the original culture did not consist of more than one strain. One interesting aspect is that Wiegel et al. (1981) report that DNA–DNA hybridization supported the recognition of strains JW 701/3 and strain KIVU as members of the same species, but distinct from *C. thermoaceticum* (*M. thermoacetica*). Kimura et al. (2016) have previously reported a similar problem with the designated type strain of *M. thermoautotrophica.* Formulated as a Request for an Opinion, this limits any action that can be taken to a formal ruling by that body. However, their work concentrates largely on the interpretation of 16S rRNA gene sequences that appear to have been obtained by both cloning and the isolation of strains from the culture supplied. Representative partial sequences of the 16S rRNA genes of the seven groups obtained by cloning and sequencing of the isolates have been deposited as LC133084–LC133087 and designated in the publication as representing OUT-1 to OUT-4 in that order, respectively. Kimura et al. (2016) concentrate on a single 16S rRNA gene sequence deposited as L09168 (from DSM 1974) and do not mention that additional sequences are available, X58353 and X77849. X58353 (strain JW 701/3; 1155 bases, but with numerous Ns) was deposited in 1990 from the University of Kiel and will not be considered further. X77849 was deposited in 1994 from the University of Reading in co-operation with Dr. Hippe (DSMZ curator of the strain at the time) and is derived from DSM 1974 and presumably directly from stocks held in the DSMZ. L09168 was deposited in 1993 from The University of Queensland. A direct alignment of the two sequences L09168 and X77849 indicates that, ignoring a small number of Ns in X77849, the two are not identical making it difficult to conclude whether either of the two can be considered to be a 100% accurate reflection of the original gene sequences from the same strain. Similarly, a comparison with the 16S rRNA sequences from Kimura et al. (2016) also indicate that neither of the two sequences (X77849 and L09168) (Supplementary Figure S2) show 100% similarity with those obtained by Kimura et al. (2016). It should also be remembered that the sequences X77849 and L09168 are only one part of the evidence that were not obtained directly when the type strain was originally described and “verification” of X77849 vs. L09168 does not allow one to conclude that one sequence is “correct” and the other in error. If one were to extend the reasoning of Kimura et al. (2016) to other similar cases one would conclude that given the differences between the 16S rRNA gene sequence obtained by direct amplification and that extracted from the genome of *M. stamsii* that the type strain does not exist. An even more dramatic example is the case of *Alterococcus agarolyticus* (Shieh and Jean, 1998) that started its taxonomic career as an atypical member of the *Enterobacteriaceae* (Shieh and Jean, 1998) under the 16S rRNA gene sequence AF075271.1 (deposited 19^th^ June 1998) that was substituted for by AF075271.2 (deposited 21^st^ August 2002) and is widely accepted as a member of the *Verrucomicrobia*. Under these circumstances, the nomenclatural type currently available certainly does not correspond to the 16S rRNA gene sequence originally deposited as AF075271.1 and one would have to conclude that the type strain no longer exists. However, put in context other data in the original publication clearly indicates that *Alterococcus agarolyticus* was an atypical member of the *Enterobacteriaceae* and that the original 16S rRNA gene sequence AF075271.1 is in error and should have been verified.

In the case of *M. thermoautotrophica*, comparison with the 16S rRNA gene sequence deposited as X77849 and L09168 also needs to be treated with caution if the original source culture (DSM 1974^T^) was not a pure culture or where the quality/accuracy of gene sequencing technologies may have changed over the decades. No attempt was made to compare the physiological/biochemical properties of the strains studied by Kimura et al. (2016) with the original work of Wiegel et al. (1981) and relies solely on one older gene sequence (L09168) that is not corroborated by another sequence (X77849) obtained at about the same time from the same source culture, DSM 1974^T^. Examining the 16S rRNA sequences deposited by Kimura et al. (2016) (LC133084-LC133087) against L09168, X77849 and those extracted from the genomes derived from subcultures of DSM 1974 and ATCC 33924 (including re-deposits as DSM 103132 and DSM 103284), i.e., CP017019.1 (positions 154745–156300 and 147549–149104), CP017237.1 (positions 144877–146432), and VCDX01000030.1 (positions 1667–112) indicates that toward the end of the single primer amplified partial sequences LC133085 and LC133086 gaps are present that are not otherwise present in any of the other sequences in a region that could be considered to be conserved (Supplementary Figure S2). These gaps have, therefore, not been taken into consideration in the analysis here. Kimura et al. (2016) do not provide alignments of sequences in support of their work and make it impossible to determine why they consider “none of the sequences were similar to *M. thermoautotrophica* DSM 1974T (L09168),” when in fact they show only minimal differences in the alignments presented here. Although alignments are critical steps in the evaluation of sequence-based data (both nucleotide and amino acid based) they are rarely given, contrary to recommendations (Tindall et al., 2010), making the direct verification of the resulting interpretation via this critical step impossible and are therefore included in Supplementary Figures S1, S2. The sequence LC133087 appears to belong to a strain having the most similar 16S rRNA sequence to *M. humiferrea* strain 64_FGQ^T^ (GQ872425) and will not be considered further. In the alignment shown, CP017019.1 (positions 154745–156300), CP017019.1 (positions 147549–149104), VCDX01000030.1 (positions 1667–112), and LC133086.1 have a “T” at position 280 (alignment numbering, Supplementary Figure S2) while CP017237.1 (positions 144877–146432), LC133084.1, and LC133085.1 have a “C” at the same position. LC133086.1 differs from CP017019.1 (positions 154745–156300), CP017019.1 (positions 147549–149104), and VCDX01000030.1 (positions 1667–112) in having a “T” position 435 rather than a “C” that is present in all other sequences (Supplementary Figure S2). LC133084.1 appears to be identical in the aligned bases to CP017237.1 (positions 144877–146432), but LC133085.1 has an “A” at position 745 rather than a “G” that is present in all other sequences (Supplementary Figure S2). Based on these observations, the only organism recovered in this study and that of Kimura et al. (2016) is that represented by LC133084.1 and CP017237.1 (DSM 103284). While this demonstrates the care that has to be taken in evaluating the interpretation of the data used by Kimura et al. (2016), the major problem that arises centers on the fact that the strains isolated by Kimura et al. (2016) have not been deposited in a culture collection and comparison with the original physiological and biochemical data published by Wiegel et al. (1981) cannot be made. Based on an evaluation of the 16S rRNA sequences determined previously and those determined here it is not possible to conclude that the type strain no longer exists, since it was deposited as DSM 1974 and ATCC 33924 and the 16S rRNA sequences deposited as X77849 and L09168 do not appear to be fully accurate.

The Request for an Opinion of Kimura et al. (2016) also misinterprets the wording of Rule 18c and draws incorrect conclusions. Tindall (2016) provided a detailed discussion of the incorrect interpretation of Rule 18c that was also applied by Kimura et al. (2016). Based on the evidence presented by Kimura et al. (2016) and that obtained in this work one cannot conclude that the nomenclatural type no longer exists, but rather there may be an issue with the purity of the culture deposited/currently available. The current study covers the physiological/biochemical properties of strains isolated from DSM 1974^T^ and expands on the genomic characterization of the strains studied. While it is clear that DSM 103132 and DSM 103284 (both derived from DSM 1974^T^, the designated type strain of *M. thermoautotrophica*) are more appropriately considered to be members of the species *M. thermoacetica*, there is a formal nomenclatural issue that also needs to be addressed that requires reference to be made to the International Code of Nomenclature of Prokaryotes (Parker et al., 2019). Typically, the nomenclatural type of a species as defined in Rule 18a is an axenic culture, but there are instances where one component part of a syntrophic co-culture has been named and the co-culture accepted as the nomenclatural type (type strain). However, when mixed cultures or consortia are considered (see Rule 31a and 31b) and these are treated as a “single” biological entity, the names associated with them are not validly published and could be applied to *M. thermoautotrophica*. In the case of DSM 1974^T^ and ATCC 33924^T^, although the strains currently in circulation appear to be a mixed culture, there is no unambiguous evidence that the parent culture, strain JW 701/3, was also a mixed culture. In contrast to the study of Kimura et al. (2016), it has been possible to study in greater detail pure cultures of strains isolated from DSM 1974^T^ (that is the parent deposit for all other culture collection strains) and subsequently deposited as DSM 103132 and DSM 103284. In both cases, the strains appear to be members of the species *M. thermoacetica*. One possible solution would be to designate one of them as a neotype, although based on the physiological and biochemical data presented here neither of the two strains (DSM 103132 or DSM 103284) can unambiguously be shown to be more similar in its properties than the other to the data originally published by Wiegel et al. (1981). Irrespective of which course of action is taken, it is clear that the culture of DSM 1974^T^ made available to the current authors contains strains that should be classified in the species *M. thermoacetica* leading to the logical conclusion that DSM 103132 and DSM 103284 should be assigned to that species. This nomenclatural conclusion is inescapable, irrespective of whether one follows the arguments of Kimura et al. (2016), where the name *M. thermoautotrophica* would eventually be rejected, declared to not have been validly published, or whether one considers the names *M. thermoacetica* (Fontaine et al., 1942; Collins et al., 1994) and *M. thermoautotrophica* (Wiegel et al., 1981; Collins et al., 1994) to be heterotypic synonyms. In the latter case, priority is governed by Rule 23a, 38 and 42 where the dates of valid publication of the epithets are taken into consideration, i.e., *thermoacetica* Fontaine et al. (1942) has priority over *thermoautotrophica* Wiegel et al. (1981). This also leads to the use of the name *M. thermoacetica* (Fontaine et al., 1942; Collins et al., 1994) and recognition of *M. thermoautotrophica* (Wiegel et al., 1981; Collins et al., 1994) as the later heterotypic synonym when their respective nomenclatural types are considered to members of the same taxon. The current authors favor the latter course of action, but the Judicial Commission may also decide otherwise. Also, *M. thermoautotrophica* DSM 7417 should be reclassified as *M. thermoacetica* as well.

In addition to resolving the *M. thermoacetica/thermoautotrophica* problem, this comprehensive analysis of the genus *Moorella* by the study of a significant number of novel genome sequences and knowledge of phenotypic differences led to two other important conclusions. First, strain DSM 21394, currently still named *M. thermoacetica*, clearly does not belong to this species. Reclassification and renaming as a new species are required. Secondly, *M. glycerini* NMP, *M. stamsii* DSM 26217^T^, and *M. perchloratireducens* cannot be distinguished at species level. Furthermore, *M. glycerini* NMP has been wrongly assigned as *M. glycerini* as this strain shows an ANIm value of 94% similarity compared to the type strain DSM 11254^T^ and is clearly a different species despite the high 16S rRNA gene sequence pairwise similarity of 99.7%. Based on the data presented here, *M. glycerini* NMP, *M. stamsii* DSM 26217^T^, and *M. perchloratireducens* are all members of the same species. Although reclassification of these three strains may be required, caution needs to be exercised when one considers differences between the data reported here and that previously reported in the literature (Slobodkin et al., 1997; Balk et al., 2008; Alves et al., 2013), especially with regards to the 16S rRNA gene sequences and the genomic similarity inferred from DNA–DNA hybridization experiments vs. *in silico* comparisons.

## Data Availability Statement

The datasets generated for this study can be found in the IMG, GenBank, NCBI.

## Author Contributions

SR, AP, FB, TJ, CJ, PD, and AN conceived and designed the experiments. SR, AP, CE, FB, and TJ performed the experiments. SR, AP, CE, FB, TJ, CJ, PD, and AN analyzed the data. SR, AP, FB, TJ, CJ, BT, RD, PD, and AN wrote the manuscript.

## Conflict of Interest

BT was employed by company Leibniz-Institut DSMZ-Deutsche Sammlung von Mikroorganismen und Zellkulturen GmbH, Brunswick, Germany. The remaining authors declare that the research was conducted in the absence of any commercial or financial relationships that could be construed as a potential conflict of interest.

## Footnotes

1 https://github.com/ctSkennerton/minced

2 https://github.com/aleimba/bac-genomics-scripts

3 https://github.com/jvollme

4 https://github.com/widdowquinn/pyani

5 https://github.com/aleimba/bac-genomics-scripts

## Abbreviations

ANI: average nucleotide identity
ATCC: American Type Culture Collection
CRISPR: clustered regularly interspaced short palindromic repeats
DSM(Z): Deutsche Sammlung von Mikroorganismen (und Zellkulturen)
MLSA: multi locus sequence analysis
OG: orthologous groups.
