# Supplementary material for: Genome-Based Comparison of All Species of the Genus Moorella, and Status of the Species Moorella thermoacetica and Moorella thermoautotrophica
Source: Front Microbiol. 2020 Jan 17;10:3070. doi: 10.3389/fmicb.2019.03070 (PMC6978639; doi:10.3389/fmicb.2019.03070)
Supplement: Supplementary file 1 [file Image_1.pdf]

Fig. S1: Multiple 16S rRNA gene sequence alignments of the genes extracted from the genomes and the corresponding, previously PCR amplified genes of *Moorella glycerini* DSM 11254, strain NMP, *M. humiferrea* DSM 23268, *M. mulderi* DSM 14980, *M. stamsii* DSM 26217, and *M. perchloratireducens*.

Organism	Genome 16S	16S rRNA PCR
M. glycerini DSM 11254T	CP046244.1	U82327.1
M. glycerini NMP	CELZ01000013.1_36320-37869	not deposited
M. humiferrea DSM 23268T	PVXM01000026.1_c1672-113	GQ872425
M. mulderi DSM 14980T	LTBC01000042.1_44-1687	AF487538.1
M. stamsii DSM 26217T	PVXL01000051.1_179-1739	HF563589.1
M. perchloratireducens An10	Gp0011525_16S	EF060194

```
CLUSTAL W 2.0 multiple sequence alignment

CP046244.1                    --------------ACGGAGAGTTTGATCCTGGCTCAGGACAAACGCTGGCGGCGTGCCTAACACATGCA 70
U82327.1                      ----------------------------------GTTGGACAAACGCTGGCGGCGTGCCTAACACATGCA 70
CELZ01000013.1_36320-37869    ------------------AGAGTTTGATCCTGGCTCAGGACAAACGCTGGCGGCGTGCCTAACACATGCA 70
PVXM01000026.1_c1672-113      -------------CATGGAGAGTTTGATCCTGGCTCAGGACTAACGCTGGCGGCGTGCCTAACACATGCA 70
GQ872425.1                    --------------------------------------------------CGGCGTGCCTAACACATGCA 70
LTBC01000042.1_44-1687        ------------------AGAGTTTGATCCTGGCTCAGGACAAACGCTGGCGGCGTGCCTAACACATGCA 70
AF487538.1                    CACCCACGACAGCGTCGCTGCGTCAGGCTTTCGCGTTGGACCAACGCTGGCTTCGTGCCTAACAACTGCA 70
PVXL01000051.1_179-1739       ------------TTACGGAGAGTTTGATCCTGGCTCAGGACAAACGCTGGCGGCGTGCCTAACACATGCA 70
HF563589.1                    ---------------------------------------------------------------------- 70
Gp0011525_16S                 -----------------------------CTGGCTCAGGACAAACGCTGGCGGCGTGCCTAACACATGCA 70
EF060194.1                    ---------------------------------------------------------------------- 70


CP046244.1                    AGTCGAGCGGTCTTTGACCCGGCGGAATCTTCGGAGGAAGCGGGTTAAAGATAGCGGCGGACGGGTGAGT 140
U82327.1                      AGTCGAGCGGTCTTTGACCCGGCGGAATCTTCGGAGGAAGCGGGTTAAAGATAGCGGCGGACGGGTGAGT 140
CELZ01000013.1_36320-37869    AGTCGAGCGGTCTTT-ACCTTGCGGAATCTTCGGAGGAAGCGAGGTAAAGATAGCGGCGGACGGGTGAGT 140
PVXM01000026.1_c1672-113      AGTCGAGCGGTCTTT-ACCTGGTGGAATCTTCGGAGGAAGCCGGGTAGAGATAGCGGCGGACGGGTGAGT 140
GQ872425.1                    AGTCGAGCGGTCTTT-ACTTGGTGGAATCTTCGGAGGAAGCCTGGTAGAGATAGCGGCGGACGGGTGAGT 140
LTBC01000042.1_44-1687        AGTCGAGCGGTCTTT-ACTTTGCGGAATCTTCGGAGGAAGCGAGGTAAAGATAGCGGCGGACGGGTGAGT 140
AF487538.1                    AGTCGAGCGGTCTTTGACCCGGCGGAATCTTCGGAGGAAGCTTTTTAAAGATAGCGGCGGACCCGTGAGT 140
PVXL01000051.1_179-1739       AGTCGAGCGGTCTTT-ACCTTGCGGAATCTTCGGAGGAAGCGAGGTAAAGATAGCGGCGGACGGGTGAGT 140
HF563589.1                    AGTCGAGCGGTCTTT--CCTTGCGGAATCTTCGGAGGAAGCGAGGTAAAGATAGCGGCGGACGGGTGAGT 140
```

```
Gp0011525_16S                 AGTCGAGCGGTCTTT-ACCTTGCGGAATCTTCGGAGGAAGCGAGGTAAAGATAGCGGCGGACGGGTGAGT 140
EF060194.1                    -----AGCGGTCTTT-AGCGAGCGGAATCTTCGGAGGAAGCGAGTTAAAGGTAGCGGCGGACGGGTGAGT 140
                                   **********      * ******************    ** ** ***********  ******

CP046244.1                    AACGCGTGGGTAATCTACCCTTCAGACCGGGATAACACCGGGAAACTGGTGCTAATACCGGATACGTTCT 210
U82327.1                      AACGCGTGGGTAATCTACCCTTCAGACCGGGATAACACCGGGAAACTGGTGCTAATACCGGATACGTTCT 210
CELZ01000013.1_36320-37869    AACGCGTGGGTAATCTACCCTTCAGACCGGGATAACACCGGGAAACTGGTGCTAATACCGGATACGTTCT 210
PVXM01000026.1_c1672-113      AACGCGTGGGCAATCTACCCTTCAGACCGGGATAACACTGGGAAACTGGTGCTAATACCGGATACGTTCC 210
GQ872425.1                    AACGCGTGGGCAATCTACCCTTCAGACCGGGATAACACTGGGAAACTGGTGCTAATACCGGATACGTTCC 210
LTBC01000042.1_44-1687        AACGCGTGGGTAATCTACCCTTCAGACCGGGATAACACCGGGAAACTGGTGCTAATACCGGATACGTTCT 210
AF487538.1                    AACGCGTGGGTAATCTACCCTTCAGACCGGGATAACACCGGGAAACTGGTGCTAATACCGGATACGTTCT 210
PVXL01000051.1_179-1739       AACGCGTGGGTAATCTACCCTTCAGACCGGGATAACACCGGGAAACTGGTGCTAATACCGGATACGTTCT 210
HF563589.1                    AACGCGTGGGTAATCTACCCTTCAGACCGGGATAACACCGGGAAACTGGTGCTAATACCGGATACGTTCT 210
Gp0011525_16S                 AACGCGTGGGTAATCTACCCTTCAGACCGGGATAACACCGGGAAACTGGTGCTAATACCGGATACGTTCT 210
EF060194.1                    AACGCGTGGGCAATCTACCCTTCAGGCCGGGATAACACCGGGAAACTGGTGCTAATACCGGATACGGTCT 210
                              ********** ************** ************ *************************** **

CP046244.1                    GCTGGAGGCATCTCCGGTAGAAGAAAAGGGAACGTGAGTGACCGCTGAAGGATGAGCCCGCGTCCCATT- 280
U82327.1                      GCTGGAGGCATCTCCGGTAGAAGAAAAGGGAACGTGAGTGACCGCTGAAGGATGAGCCCGCGTCCCATTA 280
CELZ01000013.1_36320-37869    ACTGGAGGCATCTCCGGTAGAAGAAAAGGAAACGCGAGTGACCGCTGAAGGATGAGCCCGCGTCCCATTA 280
PVXM01000026.1_c1672-113      CTGGGAGGCATCTTCCGGGGAAGAAAAGGGAG-GCGA-CGACCGCTGAAGGATGAGCCCGCGTCCCATTA 280
GQ872425.1                    CTGGGAGGCATCTTCTGGGGAAGAAAAGGGAG-GCGA-CGACCGCTGAAGGATGAGCCCGCGTCCCATTA 280
LTBC01000042.1_44-1687        ACCGGAGGCATCTCGGGTAGAAGAAA--GGAA-GCAAGCGACCGCTGAAGGATGAGCCCGCGTCCCATTA 280
AF487538.1                    GCTGGAGGCATCTCCGGTAGAAGAAA-GGGAACGTGAGTGACCGATGAAGGATGAGTTCGCGTCCCATTA 280
PVXL01000051.1_179-1739       ACTGGAGGCATCTCCGGTAGAAGAAAAGGAAACGCGAGTGACCGCTGAAGGATGAGCCCGCGTCCCATTA 280
HF563589.1                    ACTGGAGGCATCTCCGGTAGAAGAAAAGGAAACGCGAGTGACCGCTGAAGGATGAGCCCGCGTCCCATTA 280
Gp0011525_16S                 ACTGGAGGCATCTCCGGTAGAAGAAAAGGAAACGCGAGTGACCGCTGAAGGATGAGCCCGCGTCCCATTA 280
EF060194.1                    CCGGGAGGCATCTTCTGGAGAAGAAA-GGTGGCGCAAGCTACCGCTGAAGGATGAGCCCGCGTCCCATTA 280
                                 **********   *  *******  *    *  *   **** ***********  ***********

CP046244.1                    ------------------------------------------------------------GCCGGCCACAC 350
U82327.1                      GCTAGTTGGTGAGGTAACGGCTCACCAAGGCGACGATGGGTAGCCGGCCTGAGAGGGTGGCCGGCCACAC 350
CELZ01000013.1_36320-37869    GCTAGTTGGTGAGGTAACGGCTCACCAAGGCGACGATGGGTAGCCGGCCTGAGAGGGTGGCCGGCCACAC 350
PVXM01000026.1_c1672-113      GCTAGTTGGTGAGGTAACGGCTCACCAAGGCGACGATGGGTAGCCGGCCTGAGAGGGTGGTCGGCCACAC 350
GQ872425.1                    GCTAGTTGGTGAGGTAACGGCTCACCAAGGCGACGATGGGTAGCCGGCCTGAGAGGGTGGTCGGCCACAC 350
LTBC01000042.1_44-1687        GCTAGTTGGTGAGGTAACGGCTCACCAAGGCGACGATGGGTAGCCGGCCTGAGAGGGTGGTCGGCCACAC 350
AF487538.1                    GCTAGTTGGTGAGGTAACGGCTCACCAAGGCGACGATGGGTAGCCGGCCTGAGAGGGTGGCCGGCCAGCC 350
PVXL01000051.1_179-1739       GCTAGTTGGTGAGGTAACGGCTCACCAAGGCGACGATGGGTAGCCGGCCTGAGAGGGTGGCCGGCCACAC 350
HF563589.1                    GCTAGTTGGTGAGGTAACGGCTCACCAAGGCGACGATGGGTAGCCGGCCTGAGAGGGTGGCCGGCCACAC 350
Gp0011525_16S                 GCTAGTTGGTGAGGTAACGGCTCACCAAGGCGACGATGGGTAGCCGGCCTGAGAGGGTGGCCGGCCACAC 350
```

```
EF060194.1                    GCTAGTTGGTGAGGTAACGGCCCACCAAGGCGACGATGGGTAGCCGGCCTGAGAGGGTGGCCGGCCACAC 350
                                                                                         * ******  *


CP046244.1                    TGGGACTGAGACACGGCCCAGACTCCTACGGGAGGCAGCAGTGGGGAATCTTGCGCAATGGGCGAAAGCC 420
U82327.1                      TGGGACTGAGACACGGCCCAGACTCCTACGGGAGGCAGCAGTGGGGAATCTTGCGCAATGGGCGAAAGCC 420
CELZ01000013.1_36320-37869    TGGGACTGAGACACGGCCCAGACTCCTACGGGAGGCAGCAGTGGGGAATCTTGCGCAATGGGCGAAAGCC 420
PVXM01000026.1_c1672-113      TGGGACTGAGACACGGCCCAGACTCCTACGGGAGGCAGCAGTGGGGAATCTTGCGCAATGGGCGAAAGCC 420
GQ872425.1                    TGGGACTGAGACACGGCCCAGACTCCTACGGGAGGCAGCAGTGGGGAATCTTGCGCAATGGGCGAAAGCC 420
LTBC01000042.1_44-1687        TGGGACTGAGACACGGCCCAGACTCCTACGGGAGGCAGCAGTGGGGAATCTTGCGCAATGGGCGAAAGCC 420
AF487538.1                    TGGGACTGAGACACGGCCCAGACTCCTACGGGAGGCAGCAGTGGGGAATCTTGCGCAATGGGCGAAAGCC 420
PVXL01000051.1_179-1739       TGGGACTGAGACACGGCCCAGACTCCTACGGGAGGCAGCAGTGGGGAATCTTGCGCAATGGGCGAAAGCC 420
HF563589.1                    TGGGACTGAGACACGGCCCAGACTCCTACGGGAGGCAGCAGTGGGGAATCTTGCGCAATGGGCGAAAGCC 420
Gp0011525_16S                 TGGGACTGAGACACGGCCCAGACTCCTACGGGAGGCAGCAGTGGGGAATCTTGCGCAATGGGCGAAAGCC 420
EF060194.1                    TGGGACTGAGACACGGCCCAGACTCCTACGGGAGGCAGCAGTGGGGAATCTTGCGCAATGGGGGAAACCC 420
                              ************************************************************** **** **


CP046244.1                    TGACGCAGCGACGCCGCGTGAGCGATGAAGGCCTTCGGGTTGTAAAGCTCTGTCATCAGGGACGAAGTCT 490
U82327.1                      TGACGCAGCGACGCCGCGTGAGCGATGAAGGCCTTCGGGTTGTAAAGCTCTGTCATCAGGGACGAAGTCT 490
CELZ01000013.1_36320-37869    TGACGCAGCGACGCCGCGTGAGCGATGAAGGCCTTCGGGTTGTAAAGCTCTGTCATCAGGGACGAAGTCT 490
PVXM01000026.1_c1672-113      TGACGCAGCGACGCCGCGTGAGCGATGAAGGCCTTCGGGTCGTAAAGCTCTGTCATCAGGGACGAAGTCT 490
GQ872425.1                    TGACGCAGCGACGCCGCGTGAGCGATGAAGGCCTTCGGGTCGTAAAGCTCTGTCATCAGGGACGAAGTCT 490
LTBC01000042.1_44-1687        TGACGCAGCGACGCCGCGTGAGCGATGAAGGCCTTCGGGTTGTAAAGCTCTGTCATCAGGGACGAAGTCT 490
AF487538.1                    TGACGTCTCGACGCCGCGTGAGTAATGAAGGCCTTCGGGTTGTAAAGCTCTGTCATCAGGGACGAAGTCT 490
PVXL01000051.1_179-1739       TGACGCAGCGACGCCGCGTGAGCGATGAAGGCCTTCGGGTTGTAAAGCTCTGTCATCAGGGACGAAGTCT 490
HF563589.1                    TGACGCAGCGACGCCGCGTGAGCGATGAAGGCCTTCGGGTTGTAAAGCTCTGTCATCAGGGACGAAGTCT 490
Gp0011525_16S                 TGACGCAGCGACGCCGCGTGAGCGATGAAGGCCTTCGGGTTGTAAAGCTCTGTCATCAGGGACGAAGTCT 490
EF060194.1                    TGACGCAGCAACGCCGCGTGAGCGATGAAGGCCTTCGGGTTGTAAAGCTCTGTCATCAGGGACGAAGTCT 490
                              *****   * ************  **************** *****************************

CP046244.1                    C----GATAAG---------AGGTGACGGTACCTGAGGAGGAAGCCCCGGCTAACTACGTGCCAGCAGCC 560
U82327.1                      C----GATAAG---------AGGTGACGGTACCTGAGGAGGAAGCCCCGGCTAACTACGTGCCAGCAGCC 560
CELZ01000013.1_36320-37869    C--TGGATAAG---------AGGTGACGGTACCTGAGGAGGAAGCCCCGGCTAACTACGTGCCAGCAGCC 560
PVXM01000026.1_c1672-113      CGCTTTAGGCG---------AGGTGACGGTACCTGAAGAGGAAGCCCCGGCTAACTACGTGCCAGCAGCC 560
GQ872425.1                    CGCTT-AGGCG---------AGGTGACGGTACCTGAAGAGGAAGCCCCGGCTAACTACGTGCCAGCAGCC 560
LTBC01000042.1_44-1687        CG-TGCAAACG---------AGGTGACGGTACCTGAGGAGGAAGCCCCGGCTAACTACGTGCCAGCAGCC 560
AF487538.1                    C---G-ATAAG---------AGGTGACGGTACCTGAGGAGGAAGCCCCCCCTAACTACGTGCCAGCAGCC 560
PVXL01000051.1_179-1739       C--TGGATAAG---------AGGTGACGGTACCTGAGGAGGAAGCCCCGGCTAACTACGTGCCAGCAGCC 560
HF563589.1                    C--TGGATAAG---------AGGTGACGGTACCTGAGGAGGAAGCCCCGGCTAACTACGTGCCAGCAGCC 560
```

```
Gp0011525_16S                   C--TGGATAAG----------AGGTGACGGTACCTGAGGAGGAAGCCCCGGCTAACTACGTGCCAGCAGCC 560
EF060194.1                      TA-AAGGCGAATAGTCTTTAAGGTGACGGTACCTGAGGAGGAAGCCCCGGCTAACTACGTGCCAGCAGCC 560
                                                    **************** ***********  ********************


CP046244.1                      GCGGTAAAACGTAGGGGGCGAGCGTTGTCCGGAATTACTGGGCGTAAAGGGCGTGTAGGCGGCCCGGCAA 630
U82327.1                        GCGGTAAAACGTAGGGGGCGAGCGTTGTCCGGAATTACTGGGCGTAAAGGGCGTGTAGGCGGCCCGGCAA 630
CELZ01000013.1_36320-37869      GCGGTAAAACGTAGGGGGCGAGCGTTGTCCGGAATTACTGGGCGTAAAGGGCGTGTAGGCGGCCCGGCAA 630
PVXM01000026.1_c1672-113        GCGGTAAGACGTAGGGGGCGAGCGTTGTCCGGAATTACTGGGCGTAAAGGGCGTGTAGGCGGCCTGGCAA 630
GQ872425.1                      GCGGTAAGACGTAGGGGGCGAGCGTTGTCCGGAATTACTGGGCGTAAAGGGCGTGTAGGCGGCCTGGCAA 630
LTBC01000042.1_44-1687          GCGGTAAAACGTAGGGGGCGAGCGTTGTCCGGAATTACTGGGCGTAAAGGGCGTGTAGGCGGCCCGGCAA 630
AF487538.1                      TTGGTAAAACGTAGGGGGCGAGCGTTGTCCGGAATTACTGGGCGTAAAGGGCGTGTAGGCGGCCCGGCAA 630
PVXL01000051.1_179-1739         GCGGTAAAACGTAGGGGGCGAGCGTTGTCCGGAATTACTGGGCGTAAAGGGCGTGTAGGCGGCCCGGCAA 630
HF563589.1                      GCGGTAAAACGTAGGGGGCGAGCGTTGTCCGGAATTACTGGGCGTAAAGGGCGTGTAGGCGGCCCGGCAA 630
Gp0011525_16S                   GCGGTAAAACGTAGGGGGCGAGCGTTGTCCGGAATTACTGGGCGTAAAGGGCGTGTAGGCGGCCCGGCAA 630
EF060194.1                      GCGGTAAAACGTAGGGGGCGAGCGTTGTCCGGAATTACTGGGCGTAAAGGGCGTGTAGGCGGCCTGGCAA 630
                                  ***** ******************************************************** *****

CP046244.1                      GTCAGATGTGAAAAACCCAGGCTCAACCTGGGGGGTGCATTTGAAACTGGCGGGCTTGAGGGCAGGAGAG 700
U82327.1                        GTCAGATGTGAAAAACCCAGGCTCAACCTGGGGGGTGCATTTGAAACTGGCGGGCTTGAGGGCAGGAGAG 700
CELZ01000013.1_36320-37869      GTCAGATGTGAAAAACCCAGGCTCAACCTGGGGGGTGCATTTGAAACTGGCGGGCTTGAGGGCAGGAGAG 700
PVXM01000026.1_c1672-113        GTCAGATGTGAAAAACCCAGGCTCAACCTGGGGGATGCATTTGAAACTGCCGGGCTTGAGGGCAGGAGAG 700
GQ872425.1                      GTCAGATGTGAAAAACCCAGGCTCAACCTGGGGGGTGCATTTGAAACTGCCGGGCTTGAGGGCAGGAGAG 700
LTBC01000042.1_44-1687          GTCAGATGTGAAAAACCCAGGCTCAACCTGGGGGGTGCATTTGAAACTGGCGGGCTTGAGGGCAGGAGAG 700
AF487538.1                      GTCAGATGTGAAAAACCCAGGCTCAACCTGGGGGGTGCACCCGAAACTGGCGGGCGTGAGGGCAGGAGAG 700
PVXL01000051.1_179-1739         GTCAGATGTGAAAAACCCAGGCTCAACCTGGGGGGTGCATTTGAAACTGGCGGGCTTGAGGGCAGGAGAG 700
HF563589.1                      GTCAGATGTGAAAAACCCAGGCTCAACCTGGGGGGTGCATTTGAAACTGGCGGGCTTGAGGGCAGGAGAG 700
Gp0011525_16S                   GTCAGATGTGAAAAACCCAGGCTCAACCTGGGGGGTGCATTTGAAACTGGCGGGCTTGAGGGCAGGAGAG 700
EF060194.1                      GTCAGATGTGAAAAACCCCGGCTTAACCGGGGGCATGCATTTGAAACTGTCAGGCTTGAGGGCAGGAGAG 700
                                ****************** **** **** ****  ****   ******* * *** **************

CP046244.1                      GAGAGTGGAATTCCCGGTGTAGCGGTGAAATGCGTAGATATCGGGAGGAACACCAGTGGCGAAGGCGACT 770
U82327.1                        GAGAGTGGAATTCCCGGTGTAGCGGTGAAATGCGTAGATATCGGGAGGAACACCAGTGGCGAAGGCGACT 770
CELZ01000013.1_36320-37869      GAGAGTGGAATTCCCGGTGTAGCGGTGAAATGCGTAGATATCGGGAGGAACACCAGTGGCGAAGGCGACT 770
PVXM01000026.1_c1672-113        GAGAGCGGAATTCCCGGTGTAGCGGTGAAATGCGTAGATATCGGGAGGAACACCAGTGGCGAAGGCGGCT 770
GQ872425.1                      GAGAGCGGAATTCCCGGTGTAGCGGTGAAATGCGTAGATATCGGGAGGAACACCAGTGGCGAAGGCGGCT 770
LTBC01000042.1_44-1687          GAGAGTGGAATTCCCGGTGTAGCGGTGAAATGCGTAGATATCGGGAGGAACACCAGTGGCGAAGGCGACT 770
AF487538.1                      GAGAGTGGAATTCCCGGTGTAGCGGTGAAATGCGTAGATATCGGGAGGAACACCAGTGGCGAAGGCGACT 770
PVXL01000051.1_179-1739         GAGAGTGGAATTCCCGGTGTAGCGGTGAAATGCGTAGATATCGGGAGGAACACCAGTGGCGAAGGCGACT 770
HF563589.1                      GAGAGTGGAATTCCCGGTGTAGCGGTGAAATGCGTAGATATCGGGAGGAACACCAGTGGCGAAGGCGACT 770
```

```
Gp0011525_16S                  GAGAGTGGAATTCCCGGTGTAGCGGTGAAATGCGTAGATATCGGGAGGAACACCAGTGGCGAAGGCGACT 770
EF060194.1                     GAGAGTGGAATTCCCGGTGTAGCGGTGAAATGCGTAGATATCGGGAGGAACACCAGTGGCGAAGGCGACT 770
                               ***** ************************************************************* **

CP046244.1                     CTCTGGACTGACCCTGACGCTGAGGCGCGAAAGCGTGGGGAGCAAACAGGATTAGATACCCTGGTAGTCC 840
U82327.1                       CTCTGGACTGACCCTGACGCTGAGGCGCGAAAGCGTGGGGAGCAAACAGGATTAGATACCCTGGTAGTCC 840
CELZ01000013.1_36320-37869     CTCTGGACTGACCCTGACGCTGAGGCGCGAAAGCGTGGGGAGCAAACAGGATTAGATACCCTGGTAGTCC 840
PVXM01000026.1_c1672-113       CTCTGGACTGTACCTGACGCTGAGGCGCGAAAGCGTGGGGAGCAAACAGGATTAGATACCCTGGTAGTCC 840
GQ872425.1                     CTCTGGACTGTACCTGACGCTGAGGCGCGAAAGCGTGGGGAGCAAACAGGATTAGATACCCTGGTAGTCC 840
LTBC01000042.1_44-1687         CTCTGGACTGACCCTGACGCTGAGGCGCGAAAGCGTGGGGAGCAAACAGGATTAGATACCCTGGTAGTCC 840
AF487538.1                     CTCTGGACTGACCCTGACGCTGAGGGCCGAAAGCGTGGGGAGCATACAGGATTAGATACCCTGGTAGTCC 840
PVXL01000051.1_179-1739        CTCTGGACTGACCCTGACGCTGAGGCGCGAAAGCGTGGGGAGCAAACAGGATTAGATACCCTGGTAGTCC 840
HF563589.1                     CTCTGGACTGACCCTGACGCTGAGGCGCGAAAGCGTGGGGAGCAAACAGGATTAGATACCCTGGTAGTCC 840
Gp0011525_16S                  CTCTGGACTGACCCTGACGCTGAGGCGCGAAAGCGTGGGGAGCAAACAGGATTAGATACCCTGGTAGTCC 840
EF060194.1                     CTCTGGCCTGGCCCTGACGCTGAGGCGCGAAAGCGTGGGGAGCAAACAGGATTAGATACCCTGGTAGTCC 840
                               ****** ***  *************  ***************** *************************

CP046244.1                     ACGCCGTAAACGATGGGTACTAGGTGTTGGAGGTATCGACCCCTCCAGTGCCGCAGTTAACACAATAAGT 910
U82327.1                       ACGCCGTAAACGATGGGTACTAGGTGTTGGAGGTATCGACCCCTCCAGTGCCGCAGTTAACACAATAAGT 910
CELZ01000013.1_36320-37869     ACGCCGTAAACGATGGGTACTAGGTGTTGGAGGTATCGACCCCTCCAGTGCCGCAGTTAACACAATAAGT 910
PVXM01000026.1_c1672-113       ACGCCGTAAACGATGGGTACTAGGTGTTGGAGGTATCGACCCCTCCAGTGCCGCAGTTAACACAATAAGT 910
GQ872425.1                     ACGCCGTAAACGATGGGTACTAGGTGTTGGAGGTATCGACCCCTCCAGTGCCGCAGTTAACACAATAAGT 910
LTBC01000042.1_44-1687         ACGCCGTAAACGATGGGTACTAGGTGTTGGAGGTATCGACCCCTCCAGTGCCGCAGTTAACACAATAAGT 910
AF487538.1                     ACGCCGTAAACGATGGGTACTAGGTGTTGGAGGTATCGACCCCTCCAGTGCCGCAGTTAACACAATCAGT 910
PVXL01000051.1_179-1739        ACGCCGTAAACGATGGGTACTAGGTGTTGGAGGTATCGACCCCTCCAGTGCCGCAGTTAACACAATAAGT 910
HF563589.1                     ACGCCGTAAACGATGGGTACTAGGTGTTGGAGGTATCGACCCCTCCAGTGCCGCAGTTAACACAATAAGT 910
Gp0011525_16S                  ACGCCGTAAACGATGGGTACTAGGTGTTGGAGGTATCGACCCCTCCAGTGCCGCAGTTAACACAATAAGT 910
EF060194.1                     ACGCCGTAAACGATGGGTACTAGGTGTAGGAGGTATCGACCCCTTCTGTGCCGCAGTAAACACAATAAGT 910
                               *************************** **************** * ********** ******** ***

CP046244.1                     ACCCCGCCTGGGGAGTACGGCCGCAAGGCTGAAACTCAAAGGAATTGACGGGGGCCCGCACAAGCGGTGG 980
U82327.1                       ACCCCGCCTGGGGAGTACGGCCGCAAGGCTGAAACTCAAAGGAATTGACGGGGGCCCGCACAAGCGGTGG 980
CELZ01000013.1_36320-37869     ACCCCGCCTGGGGAGTACGGCCGCAAGGCTGAAACTCAAAGGAATTGACGGGGGCCCGCACAAGCGGTGG 980
PVXM01000026.1_c1672-113       ACCCCGCCTGGGGAGTACGGCCGCAAGGCTGAAACTCAAAGGAATTGACGGGGGCCCGCACAAGCGGTGG 980
GQ872425.1                     ACCCCGCCTGGGGAGTACGGCCGCAAGGCTGAAACTCAAAGGAATTGACGGGGGCCCGCACAAGCGGTGG 980
LTBC01000042.1_44-1687         ACCCCGCCTGGGGAGTACGGCCGCAAGGCTGAAACTCAAAGGAATTGACGGGGGCCCGCACAAGCGGTGG 980
AF487538.1                     ACCCCGCCTGGGGAGTCCGGCCGCAAGGCTGAAACTCAAAGGAATTGGCGGGGGCCCGCACAAGCGGTGG 980
PVXL01000051.1_179-1739        ACCCCGCCTGGGGAGTACGGCCGCAAGGCTGAAACTCAAAGGAATTGACGGGGGCCCGCACAAGCGGTGG 980
HF563589.1                     ACCCCGCCTGGGGAGTACGGCCGCAAGGCTGAAACTCAAAGGAATTGACGGGGGCCCGCACAAGCGGTGG 980
Gp0011525_16S                  ACCCCGCCTGGGGAGTACGGCCGCAAGGCTGAAACTCAAAGGAATTGACGGGGGCCCGCACAAGCGGTGG 980
```

```
EF060194.1                    ACCCCGCCTGGGGAGTACGGCCGCAAGGCTGAAACTCAAAGGAATTGACGGGGGCCCGCACAAGCGGTGG 980
                              **************** ****************************** **********************


CP046244.1                    AGCATGTGGTTTAATTCGACGCAACGCGAAGAA-CCTTACCGGGGTTTGACATCCCGCGAACC-TGGTGG 1050
U82327.1                      AGCATGTGGTTTAATTCGACGCAACGCGAAGAA-CCTTACCGGGGTTTGACATCCCGCGAACC-TGGTGG 1050
CELZ01000013.1_36320-37869    AGCATGTGGTTTAATTCGACGCAACGCGAAGAA-CCTTACCGGGGTTTGACATCCCGCGAACC-TGGTGG 1050
PVXM01000026.1_c1672-113      AGCATGTGGTTTAATTCGACGCAACGCGAAGAA-CCTTACCGGGGTTTGACATCCCGCGAACC-TGGTGG 1050
GQ872425.1                    AGCATGTGGTTTAATTCGACGCAACGCGAAGAA-CCTTACCGGGGTTTGACATCCCGCGAACC-TGGTGG 1050
LTBC01000042.1_44-1687        AGCATGTGGTTTAATTCGACGCAACGCGAAGAA-CCTTACCGGGGTTTGACATCCCGCGAACC-TGGTGG 1050
AF487538.1                    AGCATGTGGTTTAATTCGACGCAACGCGAAGAA-CCTTACCGGGGTGTGACATCCCGCGAACC-TGGTGG 1050
PVXL01000051.1_179-1739       AGCATGTGGTTTAATTCGACGCAACGCGAAGAA-CCTTACCGGGGTTTGACATCCCGCGAACC-TGGTGG 1050
HF563589.1                    AACATGTGGTTTAATTCGACGCAACGCGAAAAAACCTTACCGGGGTTTGACATCCCGCGAACCCTGGTGG 1050
Gp0011525_16S                 AGCATGTGGTTTAATTCGACGCAACGCGAAGAA-CCTTACCGGGGTTTGACATCCCGCGAACC-TGGTGG 1050
EF060194.1                    AGCATGTGGTTTAATTCGACGCAACGCGAAGAA-CCTTACCGGGGTTTGACATCCTGCGAACC-CTCTGG 1050
                              * **************************** ** ************ ******** *******    ***

CP046244.1                    AAACACCGGGGTG-CCGGTTTT-ACCGG-AGCGCGGAGA-CAGGTGGTGC-ATGGTTGTCGTCAGCTCGT 1120
U82327.1                      AAACACCGGGGTG-CCGGTTTT-ACCGG-AGCGCGGAGA-CAGGTGGTGC-ATGGTTGTCGTCAGCTCGT 1120
CELZ01000013.1_36320-37869    AAACACCGGGGTG-CCGGTTTT-ACCGG-AGCGCGGAGA-CAGGTGGTGC-ATGGTTGTCGTCAGCTCGT 1120
PVXM01000026.1_c1672-113      AAACACTGGGGTG-CCGGTTTT-ACCGG-AACGCGGAGA-CAGGTGGTGC-ATGGTTGTCGTCAGCTCGT 1120
GQ872425.1                    AAACACTGGGGTG-CCGGTTTT-ACCGG-AACGCGGAGA-CAGGTGGTGC-ATGGTTGTCGTCAGCTCGT 1120
LTBC01000042.1_44-1687        AAACACCGGGGTG-CCGGTTTTTACCGG-AGCGCGGAGA-CAGGTGGTGC-ATGGTTGTCGTCAGCTCGT 1120
AF487538.1                    AAACACCGGCGTG-CCGGTTTT-ACCGG-AGCGCGGAGA-CAGGTGGTGC-ATCGTTGTCGTCAGCTCGT 1120
PVXL01000051.1_179-1739       AAACACCGGGGTG-CCGGTTTT-ACCGG-AGCGCGGAGA-CAGGTGGTGC-ATGGTTGTCGTCAGCTCGT 1120
HF563589.1                    AAACACCGGGGTGGCCGGTTTT-ACCGGGAACGCGGAAAACAGGTGGTGCCATGGTTGTCCTCAGCTCGG 1120
Gp0011525_16S                 AAACACCGGGGTG-CCGGTTTT-ACCGG-AGCGCGGAGA-CAGGTGGTGC-ATGGTTGTCGTCAGCTCGT 1120
EF060194.1                    AAACAGGGGGGTG-CCC---TT--CGGGGAGCGCAGAGA-CAGGTGGTGC-ATGGTTGTCGTCAGCTCGT 1120
                              *****  ** *** **    **  * ** * *** ** * ********** ** ****** ********

CP046244.1                    GTCGTGAGATGTTGGGTTAAGT-CCCGCAA-CGAGCGCAACCC-TTACCTTT-AGTTGCCAGCACGTGAA 1190
U82327.1                      GTCGTGAGATGTTGGGTTAAGT-CCCGCAA-CGAGCGCAACCC-TTACCTTT-AGTTGCCAGCACGTGAA 1190
CELZ01000013.1_36320-37869    GTCGTGAGATGTTGGGTTAAGT-CCCGCAA-CGAGCGCAACCC-CTACCTTT-AGTTGCCAGCACGTAAA 1190
PVXM01000026.1_c1672-113      GTCGTGAGATGTTGGGTTAAGT-CCCGCAA-CGAGCGCAACCC-CTACCTTT-AGTTGCCAGCACGTAAT 1190
GQ872425.1                    GTCGTGAGATGTTGGGTTAAGT-CCCGCAA-CGAGCGCAACCC-CTACCTTT-AGTTGCCAGCACGTAAA 1190
LTBC01000042.1_44-1687        GTCGTGAGATGTTGGGTTAAGT-CCCGCAA-CGAGCGCAACCC-CTACCTTT-AGTTGCCAGCACGTAAA 1190
AF487538.1                    GTCGTGAGATGTTGGGTTAAGT-CCCGCAA-CGAGCGCAACCC-TTACCTTT-AGTTACCAGCACGTGAA 1190
PVXL01000051.1_179-1739       GTCGTGAGATGTTGGGTTAAGT-CCCGCAA-CGAGCGCAACCC-CTACCTTT-AGTTGCCAGCACGTAAA 1190
HF563589.1                    GTCCTGAGAAGTTGGGTTAAATTCCCGCAAACGAGCGCAACCCCTTACCTTTTAGTTGCCAGCACGTAAA 1190
Gp0011525_16S                 GTCGTGAGATGTTGGGTTAAGT-CCCGCAA-CGAGCGCAACCC-CTACCTTT-AGTTGCCAGCACGTAAA 1190
```

```
EF060194.1                     GTCGTGAGATGTTGGGTTAAGT-CCCGCAA-CGAGCGCAACCC-CTACTTTT-AGTTGCCAGCGGTAAA 1190
                               *** ***** ********** * ******* ************  *** *** **** *****   ** *


CP046244.1                     GGTGGGCACTCTAAAGGGACTGCCGGTGACAAACCGGAGGAAGGTGGGGATGACGTCAAATCATCATGCC 1260
U82327.1                       GGTGGGCACTCTAAAGGGACTGCCGGTGACAAACCGGAGGAAGGTGGGGATGACGTCAAATCATCATGCC 1260
CELZ01000013.1_36320-37869     GGTGGGCACTCTAAAGGGACTGCCGGTGACAAACCGGAGGAAGGTGGGGATGACGTCAAATCATCATGCC 1260
PVXM01000026.1_c1672-113       GGTGGGCACTCTAAAGGGACTGCCGGTGACAAACCGGAGGAAGGTGGGGATGACGTCAAATCATCATGCC 1260
GQ872425.1                     GGTGGGCACTCTAAAGGGACTGCCGGTGACAAACCGGAGGAAGGTGGGGATGACGTCAAATCATCATGCC 1260
LTBC01000042.1_44-1687         GGTGGGCACTCTAAAGGGACTGCCGGTGACAAACCGGAGGAAGGTGGGGATGACGTCAAATCATCATGCC 1260
AF487538.1                     GGTGGGCACTCTAAAGGGACTGCCGGTGACAAACCGGAGGAAGGTGGGGATGACGTCAAATCATCATGCC 1260
PVXL01000051.1_179-1739        GGTGGGCACTCTAAAGGGACTGCCGGTGACAAACCGGAGGAAGGTGGGGATGACGTCAAATCATCATGCC 1260
HF563589.1                     GGTGGGCACTCTAAAGGGACTGCCGGTGACAAACCGGAGGAAGGTGGGGATGACGTCAAATCATCATGCC 1260
Gp0011525_16S                  GGTGGGCACTCTAAAGGGACTGCCGGTGACAAACCGGAGGAAGGTGGGGATGACGTCAAATCATCATGCC 1260
EF060194.1                     GCCGGGCACTCTAAAGGGACTGCCGGTGACAAACCGGAGGAAGGTGGGGATGACGTCAAATCATCATGCC 1260
                               *  *******************************************************************

CP046244.1                     CCTTATATCCCGGGCTACACACGTGCTACAATGGCCTGTACAAAGGGGGGCGAACCCGCGAGGGGGAGCA 1330
U82327.1                       CCTTATATCCCGGGCTACACACGTGCTACAATGGCCTGTACAAAGGGGGGCGAACCCGCGAGGGGGAGCA 1330
CELZ01000013.1_36320-37869     CCTTATATCCCGGGCTACACACGTGCTACAATGGCCTGTACAAAGGGGGGCGAACCCGCGAGGGGGAGCA 1330
PVXM01000026.1_c1672-113       CCTTATATCCCGGGCTACACACGTGCTACAATGGCCGGTACAGAGGGGGGCGAACCCGCGAGGGGGAGCA 1330
GQ872425.1                     CCTTATATCCCGGGCTACACACGTGCTACAATGGCCGGTACAGAGGGGGGCGAACCCGCGAGGGGGAGCA 1330
LTBC01000042.1_44-1687         CCTTATATCCCGGGCTACACACGTGCTACAATGGCCTGTACAAAGGGGGGCGAACCCGCGAGGGGGAGCA 1330
AF487538.1                     CCTTATATCCCGGGCTACACACGTGCTACAATGGCCTGTACATTGGGGGGCGAACCCGCGAGGGGGAGCA 1330
PVXL01000051.1_179-1739        CCTTATATCCCGGGCTACACACGTGCTACAATGGCCTGTACAAAGGGGGGCGAACCCGCGAGGGGGAGCA 1330
HF563589.1                     CCTTATATCCCGGGCTACACACGTGCTACAATGGCCTGTACAAAGGGGGGCGAACCCGCGAGGGGGAGCA 1330
Gp0011525_16S                  CCTTATATCCCGGGCTACACACGTGCTACAATGGCCTGTACAAAGGGGGGCGAACCCGCGAGGGGGAGCA 1330
EF060194.1                     CCTTATATCCCGGGCTACACACGTGCTACAATGGCCTGTACAGAGGGAGGCGAAGGAGCGATCCGGAGCG 1330
                               ************************************ *****  *** ******   ****   *****

CP046244.1                     AATCCCAAAAAGCAGGTCTCAGTTCGGATTGCAGGCTGCAACTCGCCTGCATGAAGTCGGAATCGCTAGT 1400
U82327.1                       AATCCCAAAAAGCAGGTCTCAGTTCGGATTGCAGGCTGCAACTCGCCTGCATGAAGTCGGAATCGCTAGT 1400
CELZ01000013.1_36320-37869     AATCCCAAAAAGCAGGTCTCAGTTCGGATTGCAGGCTGCAACTCGCCTGCATGAAGTCGGAATCGCTAGT 1400
PVXM01000026.1_c1672-113       AATCCCAAAAAGCCGGTCTCAGTTCGGATTGCAGGCTGCAACTCGCCTGCATGAAGGCGGAATCGCTAGT 1400
GQ872425.1                     AATCCCAAAAAGCCGGTCTCAGTTCGGATTGCAGGCTGCAACTCGCCTGCATGAAGGCGGAATCGCTAGT 1400
LTBC01000042.1_44-1687         AATCCCAAAAAGCAGGTCTCAGTTCGGATTGCAGGCTGCAACTCGCCTGCATGAAGTCGGAATCGCTAGT 1400
AF487538.1                     AACTCCAAACAGCAGGTCTCAGTTCGGATTGCAGGCTGCAACTCGCCTGCATGAAGTCGGAATCGCTAGT 1400
PVXL01000051.1_179-1739        AATCCCAAAAAGCAGGTCTCAGTTCGGATTGCAGGCTGCAACTCGCCTGCATGAAGTCGGAATCGCTAGT 1400
HF563589.1                     AATCCCAAAAAGCAGGTCTCAGTTCGGATTGCAGGCTGCAACTCGCCTGCATGAAGTCGGAATCGCTAGT 1400
Gp0011525_16S                  AATCCCAAAAAGCAGGTCTCAGTTCGGATTGCAGGCTGCAACTCGCCTGCATGAAGTCGGAATCGCTAGT 1400
```

```
EF060194.1                    AATCCCAAAAAGCAGGTCTAAGTTCGGATTGCAGGCTGCAACTCGCCTGCATGAAGTCGGAATCGCTAGT 1400
                              **  ***** *** ***** ************************************ *************

CP046244.1                    AATCGCGGATCAGCATGCCGCGGTGAATACGTTCCCGGGCCTTGTACACACCGCCCGTCACACCACGAAA 1470
U82327.1                      AATCGCGGATCAGCATGCCGCGGTGAATACGTTCCCGGGCCTTGTACACACCGCCCGTCACACCACGAAA 1470
CELZ01000013.1_36320-37869    AATCGCGGATCAGCATGCCGCGGTGAATACGTTCCCGGGCCTTGTACACACCGCCCGTCACACCACGAAA 1470
PVXM01000026.1_c1672-113      AATCGCGGATCAGCATGCCGCGGTGAATACGTTCCCGGGCCTTGTACACACCGCCCGTCACACCACGAAA 1470
GQ872425.1                    AATCGCGGATCAGCATGCCGCGGTGAATACGTTCCCGGGCCTTGTACACACCGCCCGTCACACCACGAAA 1470
LTBC01000042.1_44-1687        AATCGCGGATCAGCATGCCGCGGTGAATACGTTCCCGGGCCTTGTACACACCGCCCGTCACACCACGAAA 1470
AF487538.1                    AATCGCGGATCAGCATGCCGCGGTGAATACGTTCCCGGGCCTTGTACACACCGCCCGTCACACCACGAAA 1470
PVXL01000051.1_179-1739       AATCGCGGATCAGCATGCCGCGGTGAATACGTTCCCGGGCCTTGTACACACCGCCCGTCACACCACGAAA 1470
HF563589.1                    AATCGCGGATCAGCATGCCGCGGTGAATACGTTCCCGGGCCTTGT-CACACCGCCCGTCACACCACGAAA 1470
Gp0011525_16S                 AATCGCGGATCAGCATGCCGCGGTGAATACGTTCCCGGGCCTTGTACACACCGCCCGTCACACCACGAAA 1470
EF060194.1                    AATCGCGGATCAGCATGCCGCGGTGAATACGTTCCCGGGCCTTGTACACACCGCCCGTCACACCACGAAA 1470
                              ********************************************* ************************

CP046244.1                    GCTGGCAACACCCGAAGCCGGTGACCCAACCCGCGA---------------------------------- 1540
U82327.1                      GCTGGCAACACCCGAAGCCGGTGACCCAACCCGCGA---------------------------------- 1540
CELZ01000013.1_36320-37869    GCTGGCAACACCCGAAGCCGGTGACCCAACCTGCAA---------------------------------- 1540
PVXM01000026.1_c1672-113      GCTGGCAACACCCGAAGCCGGTGACCCAACCTGAAA---------------------------------- 1540
GQ872425.1                    GCTGGCAACACCCGAAGCCGGTGACCCAACCCGCGA---------------------------------- 1540
LTBC01000042.1_44-1687        GCTGGCAACACCCGAAGCCGGTGACCCAACCCAGCACTCAACTGAAAGAGTCAGAGGAGGGTTTAAGGAG 1540
AF487538.1                    GCTGGCAACACCCGAACGCGGTGACCCAACCCGCGA---------------------------------- 1540
PVXL01000051.1_179-1739       GCTGGCAACACCCGAAGCCGGTGACCCAACCTGCAA---------------------------------- 1540
HF563589.1                    GCTGGCAACACCCGAAGCC-GTGACCCAACC-GCAA---------------------------------- 1540
Gp0011525_16S                 GCTGGCAACACCCGAAGCCGGTGA---------------------------------------------- 1540
EF060194.1                    GCTGGCAACACCCGAAGCCGGTG----------------------------------------------- 1540
                              ****************  * ***

CP046244.1                    -------------------------------------------------------------GGGAGGGAG 1610
U82327.1                      -------------------------------------------------------------GGGAGGGA- 1610
CELZ01000013.1_36320-37869    -------------------------------------------------------------AGGAGGGAG 1610
PVXM01000026.1_c1672-113      -------------------------------------------------------------AGGAGGGAG 1610
GQ872425.1                    -------------------------------------------------------------GGGAGGGAG 1610
LTBC01000042.1_44-1687        CAGGAGGAGTTCCGAGCAGGAACGAAAGCAAACCCGAAGATGACTCTTTGAGTTGAGTGCTGGGAGGGAG 1610
AF487538.1                    -------------------------------------------------------------GGGAGGGA- 1610
PVXL01000051.1_179-1739       -------------------------------------------------------------AGGAGGGAG 1610
HF563589.1                    -------------------------------------------------------------AGGAGG-AG 1610
Gp0011525_16S                 ---------------------------------------------------------------------- 1610
```

```
EF060194.1                   ---------------------------------------------------------------------- 1610


CP046244.1                   CCGTCGAAGGTGGGGCTGGTGATTGGGGTGAAGTCGTAACAAGGTAGCCGTATCGGAAGGTGCGGCTGGA 1680
U82327.1                     CCGTCCAAGGTGGGGCTGGTGATTGGGGTGAAGTCGTAACAAGGTAACCCGGGCGCC------------- 1680
CELZ01000013.1_36320-37869   CCGTCGAAGGTGGGGCTGGTGATTGGGGTGAAGTCGTAACAAGGTAGCCGTATCGGAAGGTGCGGCTGGA 1680
PVXM01000026.1_c1672-113     CCGTCGAAGGTGGGGCTGGTGATTGGGGTGAAGTCGTAACAAGGTAGCCGTATCGGAAGGTGCGGCTGGA 1680
GQ872425.1                   CCGTCTAAGGTGGGGCTGGTGATTGGGGTGAAGTCGTAACAAGGTAA----------------------- 1680
LTBC01000042.1_44-1687       CCGTCGAAGGTGGGGCTGGTGATTGGGGTGAAGTCGTAACAAGGTAGCCGTATCGGAAGGTGCGGCTGGA 1680
AF487538.1                   CCGTCCAAGGTGGGGCTGTGGATTGGGGTGAAGT------------------------------------ 1680
PVXL01000051.1_179-1739      CCGTCGAAGGTGGGGCTGGTGATTGGGGTGAAGTCGTAACAAGGTAGCCGTATCGGAAGGTGCGGCTGGA 1680
HF563589.1                   ---------------------------------------------------------------------- 1680
Gp0011525_16S                ---------------------------------------------------------------------- 1680
EF060194.1                   ---------------------------------------------------------------------- 1680

CP046244.1                   TCACCTCCTTT--------- 1700
U82327.1                     -------------------- 1700
CELZ01000013.1_36320-37869   TCACCT-------------- 1700
PVXM01000026.1_c1672-113     TCACCTCCTTT--------- 1700
GQ872425.1                   -------------------- 1700
LTBC01000042.1_44-1687       TCACCT-------------- 1700
AF487538.1                   -------------------- 1700
PVXL01000051.1_179-1739      TCACCTCCTT---------- 1700
HF563589.1                   -------------------- 1700
Gp0011525_16S                -------------------- 1700
EF060194.1                   -------------------- 1700
```

Multiple 16S rRNA gene sequence alignments of the genes extracted from the genomes and the corresponding, previously PCR amplified genes of *Moorella glycerini* DSM 11254 (CP046244.1 and U82327.1), strain NMP (CELZ01000013.1_36320-37869), *M. humiferrea* DSM 23268 (PVXM01000026.1_c1672-113 andGQ872425) *M. mulderi* DSM 14980 (LTBC01000042.1_44-1687 and AF487538.1) *M. stamsii* DSM 26217 (PVXL01000051.1_179-1739 and HF563589.1) and *M. perchloratireducens* An10 (Gp0011525_16S and EF060194). The large deletion in the 16S rRNA gene sequence from CP046244.1 is highlighted in yellow, the large insertion in LTBC01000042.1 in green and discrepancies between Gp0011525 and EF060194.1 in light blue.
