# Supplementary material for: Genome-Based Comparison of All Species of the Genus Moorella, and Status of the Species Moorella thermoacetica and Moorella thermoautotrophica
Source: Front Microbiol. 2020 Jan 17;10:3070. doi: 10.3389/fmicb.2019.03070 (PMC6978639; doi:10.3389/fmicb.2019.03070)
Supplement: Supplementary file 2 [file Image_2.pdf]

Fig. S2: Multiple 16S rRNA gene sequence alignments of the genes extracted from the genomes and the corresponding, previously PCR amplified genes of *Moorella thermoautotrophica* strains, including the representative partial 16S rRNA sequences deposited by Kimura et al. (2016) from OUT1-OTU4.

```
CLUSTAL 2.1 multiple sequence alignment

X77849.1                        -----------------------------------GGCGTGCCTAACACATG 50
L09168.1                        ---GTTTGATCCTGGCTCAGGACAAACGCTGGCGGCGTGCCTAACACATG
CP017019.1_154745-156300        AGAGTTTGATCCTGGCTCAGGACAAACGCTGGCGGCGTGCCTAACACATG
CP017019.1_147549-149104        AGAGTTTGATCCTGGCTCAGGACAAACGCTGGCGGCGTGCCTAACACATG
CP017237.1_144877-146432        AGAGTTTGATCCTGGCTCAGGACAAACGCTGGCGGCGTGCCTAACACATG
VCDX01000030.1_112-1667r        AGAGTTTGATCCTGGCTCAGGACAAACGCTGGCGGCGTGCCTAACACATG
VCDY01000019.1_112-1667r        AGAGTTTGATCCTGGCTCAGGACAAACGCTGGCGGCGTGCCTAACACATG
VCDV01000019.1_2-1557           AGAGTTTGATCCTGGCTCAGGACAAACGCTGGCGGCGTGCCTAACACATG
VCDW01000017.1_112-1667         AGAGTTTGATCCTGGCTCAGGACAAACGCTGGCGGCGTGCCTAACACATG
LC133084.1                      --------------------------------------------------
LC133085.1                      --------------------------------------------------
LC133086.1                      --------------------------------------------------
LC133087.1                      --------------------------------------------------


X77849.1                        CAAGTCGTAGCAGTCTTTAATTGGGGAAATCTTCGGATGGAACCGATTAA 100
L09168.1                        CAAGTCG-AGCGGTCTTTAATTGGGGAAATCTTCGGATGGAACCGATTAA
CP017019.1_154745-156300        CAAGTCG-AGCGGTCTTTAATTGGGGAAATCTTCGGATGGAACCGATTAA
CP017019.1_147549-149104        CAAGTCG-AGCGGTCTTTAATTGGGGAAATCTTCGGATGGAACCGATTAA
CP017237.1_144877-146432        CAAGTCG-AGCGGTCTTTAATTGGGGAAATCTTCGGATGGAACCGATTAA
VCDX01000030.1_112-1667r        CAAGTCG-AGCGGTCTTTAATTGGGGAAATCTTCGGATGGAACCGATTAA
VCDY01000019.1_112-1667r        CAAGTCG-AGCGGTCTTTAATTGGGGAAATCTTCGGATGGAACCGATTAA
VCDV01000019.1_2-1557           CAAGTCG-AGCGGTCTTTAATTGGGGAAATCTTCGGATGGAACCGATTAA
VCDW01000017.1_112-1667         CAAGTCG-AGCGGTCTTTAATTGGGGAAATCTTCGGATGGAACCGATTAA
LC133084.1                      ---GTCG-AGCGGTCTTTAATTGGGGAAATCTTCGGATGGAACCGATTAA
LC133085.1                      ---GTCG-AGCGGTCTTTAATTGGGGAAATCTTCGGATGGAACCGATTAA
LC133086.1                      ---GTCG-AGCGGTCTTTAATTGGGGAAATCTTCGGATGGAACCGATTAA
LC133087.1                      ---GTCG-AGCGGTCTTTACCTGGTGGAATCTTCGGAGGAAGCCGGGTAG
                                   **** *** *******  *** * ********** * * ***   **

X77849.1                        AGATAGCGGCCGGACGGGTGAGTAACGCGTGGGTAATCTACCCTTCAGAC 150
L09168.1                        AGATA-CCGCCGGACGGGTGAGTAACGCGTGGGTAATCTACCCTTCAGAC
CP017019.1_154745-156300        AGATAGCGGC-GGACGGGTGAGTAACGCGTGGGTAATCTACCCTTCAGAC
CP017019.1_147549-149104        AGATAGCGGC-GGACGGGTGAGTAACGCGTGGGTAATCTACCCTTCAGAC
CP017237.1_144877-146432        AGATAGCGGC-GGACGGGTGAGTAACGCGTGGGTAATCTACCCTTCAGAC
VCDX01000030.1_112-1667r        AGATAGCGGC-GGACGGGTGAGTAACGCGTGGGTAATCTACCCTTCAGAC
VCDY01000019.1_112-1667r        AGATAGCGGC-GGACGGGTGAGTAACGCGTGGGTAATCTACCCTTCAGAC
VCDV01000019.1_2-1557           AGATAGCGGC-GGACGGGTGAGTAACGCGTGGGTAATCTACCCTTCAGAC
VCDW01000017.1_112-1667         AGATAGCGGC-GGACGGGTGAGTAACGCGTGGGTAATCTACCCTTCAGAC
LC133084.1                      AGATAGCGGC-GGACGGGTGAGTAACGCGTGGGTAATCTACCCTTCAGAC
LC133085.1                      AGATAGCGGC-GGACGGGTGAGTAACGCGTGGGTAATCTACCCTTCAGAC
LC133086.1                      AGATAGCGGC-GGACGGGTGAGTAACGCGTGGGTAATCTACCCTTCAGAC
LC133087.1                      AGATAGCGGC-GGACGGGTGAGTAACGCGTGGGCAATCTACCCTTCAGAC
                                ***** * ** ********************* *****************

X77849.1                        TGGGATAACACCGGGAAACTGGTGCTAATACCGGATACGGTCTACGGGAG 200
L09168.1                        TGGGATAACACCGGGAAACTGGTGCTAATACCGGATACGGTCTACGGGAG
CP017019.1_154745-156300        TGGGATAACACCGGGAAACTGGTGCTAATACCGGATACGGTCTACGGGAG
CP017019.1_147549-149104        TGGGATAACACCGGGAAACTGGTGCTAATACCGGATACGGTCTACGGGAG
CP017237.1_144877-146432        TGGGATAACACCGGGAAACTGGTGCTAATACCGGATACGGTCTACGGGAG
VCDX01000030.1_112-1667r        TGGGATAACACCGGGAAACTGGTGCTAATACCGGATACGGTCTACGGGAG
VCDY01000019.1_112-1667r        TGGGATAACACCGGGAAACTGGTGCTAATACCGGATACGGTCTACGGGAG
VCDV01000019.1_2-1557           TGGGATAACACCGGGAAACTGGTGCTAATACCGGATACGGTCTACGGGAG
```

```
VCDW01000017.1_112-1667       TGGGATAACACCGGGAAACTGGTGCTAATACCGGATACGGTCTACGGGAG
LC133084.1                    TGGGATAACACCGGGAAACTGGTGCTAATACCGGATACGGTCTACGGGAG
LC133085.1                    TGGGATAACACCGGGAAACTGGTGCTAATACCGGATACGGTCTACGGGAG
LC133086.1                    TGGGATAACACCGGGAAACTGGTGCTAATACCGGATACGGTCTACGGGAG
LC133087.1                    CGGGATAACACTGGGAAACTGGTGCTAATACCGGATACGTTCCCTGGGAG
                               ********** *************************** **   *****

X77849.1                      GCATCTTCTGTAGAAGAAAGGTGGCGCAACGTACCGCTGAAGGATGAGCC 250
L09168.1                      GCATCTTCTGTAGAAGAAAGGTGGCGCAAGCTACCGCTGAAGGATGAGCC
CP017019.1_154745-156300      GCATCTTCTGTAGAAGAAAGGTGGCGCAAGCTACCGCTGAAGGATGAGCC
CP017019.1_147549-149104      GCATCTTCTGTAGAAGAAAGGTGGCGCAAGCTACCGCTGAAGGATGAGCC
CP017237.1_144877-146432      GCATCTTCTGTAGAAGAAAGGTGGCGCAAGCTACCGCTGAAGGATGAGCC
VCDX01000030.1_112-1667r      GCATCTTCTGTAGAAGAAAGGTGGCGCAAGCTACCGCTGAAGGATGAGCC
VCDY01000019.1_112-1667r      GCATCTTCTGTAGAAGAAAGGTGGCGCAAGCTACCGCTGAAGGATGAGCC
VCDV01000019.1_2-1557         GCATCTTCTGTAGAAGAAAGGTGGCGCAAGCTACCGCTGAAGGATGAGCC
VCDW01000017.1_112-1667       GCATCTTCTGTAGAAGAAAGGTGGCGCAAGCTACCGCTGAAGGATGAGCC
LC133084.1                    GCATCTTCTGTAGAAGAAAGGTGGCGCAAGCTACCGCTGAAGGATGAGCC
LC133085.1                    GCATCTTCTGTAGAAGAAAGGTGGCGCAAGCTACCGCTGAAGGATGAGCC
LC133086.1                    GCATCTTCTGTAGAAGAAAGGTGGCGCAAGCTACCGCTGAAGGATGAGCC
LC133087.1                    GCATCTTCCGGGGAAGAAAAGGGAGGCGA-CGACCGCTGAAGGATGAGCC
                              ******** *  ******* * *  ** *   ******************

X77849.1                      CGCGTCCCATTAGCTAGTTGGTGAGGTAATGGCTCACCAAGGCGACGATG 300
L09168.1                      CGCGTCCCATTAGCTAGTTGGTGAGGTAATGGCTCACCAAGGCGACGATG
CP017019.1_154745-156300      CGCGTCCCATTAGCTAGTTGGTGAGGTAATGGCTCACCAAGGCGACGATG
CP017019.1_147549-149104      CGCGTCCCATTAGCTAGTTGGTGAGGTAATGGCTCACCAAGGCGACGATG
CP017237.1_144877-146432      CGCGTCCCATTAGCTAGTTGGTGAGGTAACGGCTCACCAAGGCGACGATG
VCDX01000030.1_112-1667r      CGCGTCCCATTAGCTAGTTGGTGAGGTAATGGCTCACCAAGGCGACGATG
VCDY01000019.1_112-1667r      CGCGTCCCATTAGCTAGTTGGTGAGGTAACGGCTCACCAAGGCGACGATG
VCDV01000019.1_2-1557         CGCGTCCCATTAGCTAGTTGGTGAGGTAACGGCTCACCAAGGCGACGATG
VCDW01000017.1_112-1667       CGCGTCCCATTAGCTAGTTGGTGAGGTAACGGCTCACCAAGGCGACGATG
LC133084.1                    CGCGTCCCATTAGCTAGTTGGTGAGGTAACGGCTCACCAAGGCGACGATG
LC133085.1                    CGCGTCCCATTAGCTAGTTGGTGAGGTAACGGCTCACCAAGGCGACGATG
LC133086.1                    CGCGTCCCATTAGCTAGTTGGTGAGGTAATGGCTCACCAAGGCGACGATG
LC133087.1                    CGCGTCCCATTAGCTAGTTGGTGAGGTAACGGCTCACCAAGGCGACGATG
                              ***************************** ********************

X77849.1                      GGTAGCCGGCCTGAGAGGGTGGTCGGCCACACTGGGACTGAGACACGGCC 350
L09168.1                      GGTAGCCGGCCTGAGAGGGTGGTCGGCCACACTGGGACTGAGACACGGCC
CP017019.1_154745-156300      GGTAGCCGGCCTGAGAGGGTGGTCGGCCACACTGGGACTGAGACACGGCC
CP017019.1_147549-149104      GGTAGCCGGCCTGAGAGGGTGGTCGGCCACACTGGGACTGAGACACGGCC
CP017237.1_144877-146432      GGTAGCCGGCCTGAGAGGGTGGTCGGCCACACTGGGACTGAGACACGGCC
VCDX01000030.1_112-1667r      GGTAGCCGGCCTGAGAGGGTGGTCGGCCACACTGGGACTGAGACACGGCC
VCDY01000019.1_112-1667r      GGTAGCCGGCCTGAGAGGGTGGTCGGCCACACTGGGACTGAGACACGGCC
VCDV01000019.1_2-1557         GGTAGCCGGCCTGAGAGGGTGGTCGGCCACACTGGGACTGAGACACGGCC
VCDW01000017.1_112-1667       GGTAGCCGGCCTGAGAGGGTGGTCGGCCACACTGGGACTGAGACACGGCC
LC133084.1                    GGTAGCCGGCCTGAGAGGGTGGTCGGCCACACTGGGACTGAGACACGGCC
LC133085.1                    GGTAGCCGGCCTGAGAGGGTGGTCGGCCACACTGGGACTGAGACACGGCC
LC133086.1                    GGTAGCCGGCCTGAGAGGGTGGTCGGCCACACTGGGACTGAGACACGGCC
LC133087.1                    GGTAGCCGGCCTGAGAGGGTGGTCGGCCACACTGGGACTGAGACACGGCC
                              **************************************************

X77849.1                      CATACTCCTACGGGAGGCAGCAGTGGGGAATCTTGCGCAATGGGCGAAAG 400
L09168.1                      CAGACTCCTACGGGAGGCAGCAGTGGGGAATCTTGCGCAATGGGCGAAAG
CP017019.1_154745-156300      CAGACTCCTACGGGAGGCAGCAGTGGGGAATCTTGCGCAATGGGCGAAAG
CP017019.1_147549-149104      CAGACTCCTACGGGAGGCAGCAGTGGGGAATCTTGCGCAATGGGCGAAAG
CP017237.1_144877-146432      CAGACTCCTACGGGAGGCAGCAGTGGGGAATCTTGCGCAATGGGCGAAAG
VCDX01000030.1_112-1667r      CAGACTCCTACGGGAGGCAGCAGTGGGGAATCTTGCGCAATGGGCGAAAG
VCDY01000019.1_112-1667r      CAGACTCCTACGGGAGGCAGCAGTGGGGAATCTTGCGCAATGGGCGAAAG
VCDV01000019.1_2-1557         CAGACTCCTACGGGAGGCAGCAGTGGGGAATCTTGCGCAATGGGCGAAAG
VCDW01000017.1_112-1667       CAGACTCCTACGGGAGGCAGCAGTGGGGAATCTTGCGCAATGGGCGAAAG
LC133084.1                    CAGACTCCTACGGGAGGCAGCAGTGGGGAATCTTGCGCAATGGGCGAAAG
```

```
LC133085.1                    CAGACTCCTACGGGAGGCAGCAGTGGGGAATCTTGCGCAATGGGCGAAAG
LC133086.1                    CAGACTCCTACGGGAGGCAGCAGTGGGGAATCTTGCGCAATGGGCGAAAG
LC133087.1                    CAGACTCCTACGGGAGGCAGCAGTGGGGAATCTTGCGCAATGGGCGAAAG
                              ** ***********************************************

X77849.1                      CCTGACGCAGCAACGCCGCGTGAGCGATGAAGGCCTTCGGGTTGTAAAGC 450
L09168.1                      CCTGACGCAGCAACCCCGCGTGAGCGATGAAGGCCTTCGGGTTGTAAAGC
CP017019.1_154745-156300      CCTGACGCAGCAACGCCGCGTGAGCGATGAAGGCCTTCGGGTTGTAAAGC
CP017019.1_147549-149104      CCTGACGCAGCAACGCCGCGTGAGCGATGAAGGCCTTCGGGTTGTAAAGC
CP017237.1_144877-146432      CCTGACGCAGCAACGCCGCGTGAGCGATGAAGGCCTTCGGGTTGTAAAGC
VCDX01000030.1_112-1667r      CCTGACGCAGCAACGCCGCGTGAGCGATGAAGGCCTTCGGGTTGTAAAGC
VCDY01000019.1_112-1667r      CCTGACGCAGCAACGCCGCGTGAGCGATGAAGGCCTTCGGGTTGTAAAGC
VCDV01000019.1_2-1557         CCTGACGCAGCAACGCCGCGTGAGCGATGAAGGCCTTCGGGTTGTAAAGC
VCDW01000017.1_112-1667       CCTGACGCAGCAACGCCGCGTGAGCGATGAAGGCCTTCGGGTTGTAAAGC
LC133084.1                    CCTGACGCAGCAACGCCGCGTGAGCGATGAAGGCCTTCGGGTTGTAAAGC
LC133085.1                    CCTGACGCAGCAACGCCGCGTGAGCGATGAAGGCCTTCGGGTTGTAAAGC
LC133086.1                    CCTGACGCAGCAACGCCGCGTGAGCGATGAAGGCCTTCGGGTTGTAAAGC
LC133087.1                    CCTGACGCAGCGACGCCGCGTGAGCGATGAAGGCCTTCGGGTCGTAAAGC
                              *********** ** *************************** *******

X77849.1                      TCTGTCATCAGGGACGAAGTCTTAAAGGCGAATAGCCTTTAAGGTGACGG 500
L09168.1                      TCTGTCATCAGGGACGAAGTCTTAAAGGCGAATAGCCTTTAAGGTGACGG
CP017019.1_154745-156300      TCTGTCATCAGGGACGAAGTCTTAAAGGCGAATAGCCTTTAAGGTGACGG
CP017019.1_147549-149104      TCTGTCATCAGGGACGAAGTCTTAAAGGCGAATAGCCTTTAAGGTGACGG
CP017237.1_144877-146432      TCTGTCATCAGGGACGAAGTCTTAAAGGCGAATAGCCTTTAAGGTGACGG
VCDX01000030.1_112-1667r      TCTGTCATCAGGGACGAAGTCTTAAAGGCGAATAGCCTTTAAGGTGACGG
VCDY01000019.1_112-1667r      TCTGTCATCAGGGACGAAGTCTTAAAGGCGAATAGCCTTTAAGGTGACGG
VCDV01000019.1_2-1557         TCTGTCATCAGGGACGAAGTCTTAAAGGCGAATAGCCTTTAAGGTGACGG
VCDW01000017.1_112-1667       TCTGTCATCAGGGACGAAGTCTTAAAGGCGAATAGCCTTTAAGGTGACGG
LC133084.1                    TCTGTCATCAGGGACGAAGTCTTAAAGGCGAATAGCCTTTAAGGTGACGG
LC133085.1                    TCTGTCATCAGGGACGAAGTCTTAAAGGCGAATAGCCTTTAAGGTGACGG
LC133086.1                    TCTGTCATCAGGGACGAAGTCTTAAAGGCGAATAGTCTTTAAGGTGACGG
LC133087.1                    TCTGTCATCAGGGACGAAGTCTCGCT-----TTAGGC---GAGGTGACGG
                              **********************          *** *    *********

X77849.1                      TACCTGAGGAGGAAGCCCCGGCTAACTACGTGCCAGCAGCCGCGGTAAAA 550
L09168.1                      TACCTGAGGAGGAAGCCCCGGCTAACTACGTGCCAGCAGCCGCGGTAAAA
CP017019.1_154745-156300      TACCTGAGGAGGAAGCCCCGGCTAACTACGTGCCAGCAGCCGCGGTAAAA
CP017019.1_147549-149104      TACCTGAGGAGGAAGCCCCGGCTAACTACGTGCCAGCAGCCGCGGTAAAA
CP017237.1_144877-146432      TACCTGAGGAGGAAGCCCCGGCTAACTACGTGCCAGCAGCCGCGGTAAAA
VCDX01000030.1_112-1667r      TACCTGAGGAGGAAGCCCCGGCTAACTACGTGCCAGCAGCCGCGGTAAAA
VCDY01000019.1_112-1667r      TACCTGAGGAGGAAGCCCCGGCTAACTACGTGCCAGCAGCCGCGGTAAAA
VCDV01000019.1_2-1557         TACCTGAGGAGGAAGCCCCGGCTAACTACGTGCCAGCAGCCGCGGTAAAA
VCDW01000017.1_112-1667       TACCTGAGGAGGAAGCCCCGGCTAACTACGTGCCAGCAGCCGCGGTAAAA
LC133084.1                    TACCTGAGGAGGAAGCCCCGGCTAACTACGTGCCAGCAGCCGCGGTAAAA
LC133085.1                    TACCTGAGGAGGAAGCCCCGGCTAACTACGTGCCAGCAGCCGCGGTAAAA
LC133086.1                    TACCTGAGGAGGAAGCCCCGGCTAACTACGTGCCAGCAGCCGCGGTAAAA
LC133087.1                    TACCTGAAGAGGAAGCCCCGGCTAACTACGTGCCAGCAGCCGCGGTAAGA
                              ******* **************************************** *

X77849.1                      CGTAGGGGGCGAGCGTTGTCCGGAATTACTGGGCGTAAAGGGCGTGTAAG 600
L09168.1                      CGTAGGGGGCGAGCGTTGTCCGGAATTACTGGGCGTAAAGGGCGTGTA-G
CP017019.1_154745-156300      CGTAGGGGGCGAGCGTTGTCCGGAATTACTGGGCGTAAAGGGCGTGTA-G
CP017019.1_147549-149104      CGTAGGGGGCGAGCGTTGTCCGGAATTACTGGGCGTAAAGGGCGTGTA-G
CP017237.1_144877-146432      CGTAGGGGGCGAGCGTTGTCCGGAATTACTGGGCGTAAAGGGCGTGTA-G
VCDX01000030.1_112-1667r      CGTAGGGGGCGAGCGTTGTCCGGAATTACTGGGCGTAAAGGGCGTGTA-G
VCDY01000019.1_112-1667r      CGTAGGGGGCGAGCGTTGTCCGGAATTACTGGGCGTAAAGGGCGTGTA-G
VCDV01000019.1_2-1557         CGTAGGGGGCGAGCGTTGTCCGGAATTACTGGGCGTAAAGGGCGTGTA-G
VCDW01000017.1_112-1667       CGTAGGGGGCGAGCGTTGTCCGGAATTACTGGGCGTAAAGGGCGTGTA-G
LC133084.1                    CGTAGGGGGCGAGCGTTGTCCGGAATTACTGGGCGTAAAGGGCGTGTA-G
LC133085.1                    CGTAGGGGGCGAGCGTTGTCCGGAATTACTGGGCGTAAAGGGCGTGTA-G
LC133086.1                    CGTAGGGGGCGAGCGTTGTCCGGAATTACTGGGCGTAAAGGGCGTGTA-G
```

```
LC133087.1                    CGTAGGGGGCGAGCGTTGTCCGGAATTACTGGGCGTAAAGGGCGTGTA-G
                              ************************************************ *


X77849.1                      GCGGCCTGGCAAGTCAGATGTGAAAAACCCCGGCTTAACCGGGG-CATGC 650
L09168.1                      GCGGCCTGGCAAGTCAGATGTGAAAAACCCCGGCTTAACCGGGGGCATGC
CP017019.1_154745-156300      GCGGCCTGGCAAGTCAGATGTGAAAAACCCCGGCTTAACCGGGGGCATGC
CP017019.1_147549-149104      GCGGCCTGGCAAGTCAGATGTGAAAAACCCCGGCTTAACCGGGGGCATGC
CP017237.1_144877-146432      GCGGCCTGGCAAGTCAGATGTGAAAAACCCCGGCTTAACCGGGGGCATGC
VCDX01000030.1_112-1667r      GCGGCCTGGCAAGTCAGATGTGAAAAACCCCGGCTTAACCGGGGGCATGC
VCDY01000019.1_112-1667r      GCGGCCTGGCAAGTCAGATGTGAAAAACCCCGGCTTAACCGGGGGCATGC
VCDV01000019.1_2-1557         GCGGCCTGGCAAGTCAGATGTGAAAAACCCCGGCTTAACCGGGGGCATGC
VCDW01000017.1_112-1667       GCGGCCTGGCAAGTCAGATGTGAAAAACCCCGGCTTAACCGGGGGCATGC
LC133084.1                    GCGGCCTGGCAAGTCAGATGTGAAAAACCCCGGCTTAACCGGGGGCATGC
LC133085.1                    GCGGCCTGGCAAGTCAGATGTGAAAAACCCCGGCTTAACCGGGGGCATGC
LC133086.1                    GCGGCCTGGCAAGTCAGATGTGAAAAACCCCGGCTTAACCGGGGGCATGC
LC133087.1                    GCGGCCTGGCAAGTCAGATGTGAAAAACCCAGGCTCAACCTGGGGGATGC
                              ****************************** **** **** ***  ****

X77849.1                      ATTTGAAACTGAAGGGCTTGAGGGCAGGAGAGGAGAGTGGAATTCCCNGT 700
L09168.1                      ATTTGAAACTGCCGGGCTTGAGGGCAGGAGAGGAGAGTGGAATTCCCGGT
CP017019.1_154745-156300      ATTTGAAACTGCCGGGCTTGAGGGCAGGAGAGGAGAGTGGAATTCCCGGT
CP017019.1_147549-149104      ATTTGAAACTGCCGGGCTTGAGGGCAGGAGAGGAGAGTGGAATTCCCGGT
CP017237.1_144877-146432      ATTTGAAACTGCCGGGCTTGAGGGCAGGAGAGGAGAGTGGAATTCCCGGT
VCDX01000030.1_112-1667r      ATTTGAAACTGCCGGGCTTGAGGGCAGGAGAGGAGAGTGGAATTCCCGGT
VCDY01000019.1_112-1667r      ATTTGAAACTGCCGGGCTTGAGGGCAGGAGAGGAGAGTGGAATTCCCGGT
VCDV01000019.1_2-1557         ATTTGAAACTGCCGGGCTTGAGGGCAGGAGAGGAGAGTGGAATTCCCGGT
VCDW01000017.1_112-1667       ATTTGAAACTGCCGGGCTTGAGGGCAGGAGAGGAGAGTGGAATTCCCGGT
LC133084.1                    ATTTGAAACTGCCGGGCTTGAGGGCAGGAGAGGAGAGTGGAATTCCCGGT
LC133085.1                    ATTTGAAACTGCCGGGCTTGAGGGCAGGAGAGGAGAGTGGAATTCCCGGT
LC133086.1                    ATTTGAAACTGCCGGGCTTGAGGGCAGGAGAGGAGAGTGGAATTCCCGGT
LC133087.1                    ATTTGAAACTGCCGGGCTTGAGGGCAGGAGAGGAGAGCGGAATTCCCGGT
                              ***********  ************************ ********* **

X77849.1                      GTAGCGGTGAAATGCGTAGATATCGGGAGGAACACCAGTGGCGAAGGCGA 750
L09168.1                      GTAGCGGTGAAATGCGTAGATATCGGGAGGAACACCAGTGGCGAAGGCGA
CP017019.1_154745-156300      GTAGCGGTGAAATGCGTAGATATCGGGAGGAACACCAGTGGCGAAGGCGA
CP017019.1_147549-149104      GTAGCGGTGAAATGCGTAGATATCGGGAGGAACACCAGTGGCGAAGGCGA
CP017237.1_144877-146432      GTAGCGGTGAAATGCGTAGATATCGGGAGGAACACCAGTGGCGAAGGCGA
VCDX01000030.1_112-1667r      GTAGCGGTGAAATGCGTAGATATCGGGAGGAACACCAGTGGCGAAGGCGA
VCDY01000019.1_112-1667r      GTAGCGGTGAAATGCGTAGATATCGGGAGGAACACCAGTGGCGAAGGCGA
VCDV01000019.1_2-1557         GTAGCGGTGAAATGCGTAGATATCGGGAGGAACACCAGTGGCGAAGGCGA
VCDW01000017.1_112-1667       GTAGCGGTGAAATGCGTAGATATCGGGAGGAACACCAGTGGCGAAGGCGA
LC133084.1                    GTAGCGGTGAAATGCGTAGATATCGGGAGGAACACCAGTGGCGAAGGCGA
LC133085.1                    GTAGCGGTGAAATGCGTAGATATCGGGAGGAACACCAGTGGCGAAAGCGA
LC133086.1                    GTAGCGGTGAAATGCGTAGATATCGGGAGGAACACCAGTGGCGAAGGCGA
LC133087.1                    GTAGCGGTGAA-TGCGTAGATATCGGGAGGAACACCAGTGGCGAAGGCGG
                              *********** ********************************* *** 

X77849.1                      CTCTCTGGCCTGGCCCTGACGCTGAGGCGCGAAAGCGTGGGGAGCAAACA 800
L09168.1                      CTCTCTGGCCTGGCCCTGACGCTGAGGCGCGAAAGCGTGGGGAGCAAACA
CP017019.1_154745-156300      CTCTCTGGCCTGGCCCTGACGCTGAGGCGCGAAAGCGTGGGGAGCAAACA
CP017019.1_147549-149104      CTCTCTGGCCTGGCCCTGACGCTGAGGCGCGAAAGCGTGGGGAGCAAACA
CP017237.1_144877-146432      CTCTCTGGCCTGGCCCTGACGCTGAGGCGCGAAAGCGTGGGGAGCAAACA
VCDX01000030.1_112-1667r      CTCTCTGGCCTGGCCCTGACGCTGAGGCGCGAAAGCGTGGGGAGCAAACA
VCDY01000019.1_112-1667r      CTCTCTGGCCTGGCCCTGACGCTGAGGCGCGAAAGCGTGGGGAGCAAACA
VCDV01000019.1_2-1557         CTCTCTGGCCTGGCCCTGACGCTGAGGCGCGAAAGCGTGGGGAGCAAACA
VCDW01000017.1_112-1667       CTCTCTGGCCTGGCCCTGACGCTGAGGCGCGAAAGCGTGGGGAGCAAACA
LC133084.1                    CTCTCTGGCCTGGCCCTGACGCTGAGGCGCGAAAGCGTGGGGAGCAAACA
LC133085.1                    CTCTCTGGCCTGGCCCTGACGCTGAG-CGCGAAAGCGTGGGGAGCAA-CA
LC133086.1                    CTCTCTGGCCTGGCCCTGACGCTGAGGCGCGAAAGCGTGGGGAGCAA-CA
LC133087.1                    CTCTCTGGACTGTACCTGACGCTGAGGCGCGAAAGCGTGGGGAGCAAACA
```

```
                              ******** ***  ************ ******************** **

X77849.1                      GGATTAGATACCCTGGTAGTCCACGCCGTAAACGATGGGTACTAGGTGTA 850
L09168.1                      GGATTAGATACCCTGGTAGTCCACGCCGTAAACGATGGGTACTAGGTGTA
CP017019.1_154745-156300      GGATTAGATACCCTGGTAGTCCACGCCGTAAACGATGGGTACTAGGTGTA
CP017019.1_147549-149104      GGATTAGATACCCTGGTAGTCCACGCCGTAAACGATGGGTACTAGGTGTA
CP017237.1_144877-146432      GGATTAGATACCCTGGTAGTCCACGCCGTAAACGATGGGTACTAGGTGTA
VCDX01000030.1_112-1667r      GGATTAGATACCCTGGTAGTCCACGCCGTAAACGATGGGTACTAGGTGTA
VCDY01000019.1_112-1667r      GGATTAGATACCCTGGTAGTCCACGCCGTAAACGATGGGTACTAGGTGTA
VCDV01000019.1_2-1557         GGATTAGATACCCTGGTAGTCCACGCCGTAAACGATGGGTACTAGGTGTA
VCDW01000017.1_112-1667       GGATTAGATACCCTGGTAGTCCACGCCGTAAACGATGGGTACTAGGTGTA
LC133084.1                    GGATTAGATACCCTGGTAGTCCACGCC-----------------------
LC133085.1                    G-ATTAGATACCCTGGTAGTCCACGCC-----------------------
LC133086.1                    GGATTAGATACCCTGGTAGTCCACGCC-----------------------
LC133087.1                    GGATTAGATACCCTGGTAGTCCACGCC-----------------------
                              * *************************

X77849.1                      GGAGGTATNNACCCCTTCTGTGCCGCAGTAAACACAATAAGTACCCCGCC
L09168.1                      GGAGGTATCGACCCCTTGTGTGCCGCAGTAAACACAATAAGTACCCCGCC
CP017019.1_154745-156300      GGAGGTATCGACCCCTTCTGTGCCGCAGTAAACACAATAAGTACCCCGCC
CP017019.1_147549-149104      GGAGGTATCGACCCCTTCTGTGCCGCAGTAAACACAATAAGTACCCCGCC
CP017237.1_144877-146432      GGAGGTATCGACCCCTTCTGTGCCGCAGTAAACACAATAAGTACCCCGCC
VCDX01000030.1_112-1667r      GGAGGTATCGACCCCTTCTGTGCCGCAGTAAACACAATAAGTACCCCGCC
VCDY01000019.1_112-1667r      GGAGGTATCGACCCCTTCTGTGCCGCAGTAAACACAATAAGTACCCCGCC
VCDV01000019.1_2-1557         GGAGGTATCGACCCCTTCTGTGCCGCAGTAAACACAATAAGTACCCCGCC
VCDW01000017.1_112-1667       GGAGGTATCGACCCCTTCTGTGCCGCAGTAAACACAATAAGTACCCCGCC
LC133084.1                    --------------------------------------------------
LC133085.1                    --------------------------------------------------
LC133086.1                    --------------------------------------------------
LC133087.1                    --------------------------------------------------


X77849.1                      TGGGGAGTACGGCCGCAAGGCTGAAACTCAAAGGAATTGACGGGGGCCCG
L09168.1                      TGGGGAGTACGGCCGCAAGGCTGAAACTCAAAGGAATTGACGGGGGCCCG
CP017019.1_154745-156300      TGGGGAGTACGGCCGCAAGGCTGAAACTCAAAGGAATTGACGGGGGCCCG
CP017019.1_147549-149104      TGGGGAGTACGGCCGCAAGGCTGAAACTCAAAGGAATTGACGGGGGCCCG
CP017237.1_144877-146432      TGGGGAGTACGGCCGCAAGGCTGAAACTCAAAGGAATTGACGGGGGCCCG
VCDX01000030.1_112-1667r      TGGGGAGTACGGCCGCAAGGCTGAAACTCAAAGGAATTGACGGGGGCCCG
VCDY01000019.1_112-1667r      TGGGGAGTACGGCCGCAAGGCTGAAACTCAAAGGAATTGACGGGGGCCCG
VCDV01000019.1_2-1557         TGGGGAGTACGGCCGCAAGGCTGAAACTCAAAGGAATTGACGGGGGCCCG
VCDW01000017.1_112-1667       TGGGGAGTACGGCCGCAAGGCTGAAACTCAAAGGAATTGACGGGGGCCCG
LC133084.1                    --------------------------------------------------
LC133085.1                    --------------------------------------------------
LC133086.1                    --------------------------------------------------
LC133087.1                    --------------------------------------------------


X77849.1                      CACAAGCGGTGGAGCATGTGGTTTAATTCGACGCAACGCGAAGAACCTTA
L09168.1                      CACAAGCGGTGGAGCATGTGGTTTAATTCGACGCAACGCGAAGAACCTTA
CP017019.1_154745-156300      CACAAGCGGTGGAGCATGTGGTTTAATTCGACGCAACGCGAAGAACCTTA
CP017019.1_147549-149104      CACAAGCGGTGGAGCATGTGGTTTAATTCGACGCAACGCGAAGAACCTTA
CP017237.1_144877-146432      CACAAGCGGTGGAGCATGTGGTTTAATTCGACGCAACGCGAAGAACCTTA
VCDX01000030.1_112-1667r      CACAAGCGGTGGAGCATGTGGTTTAATTCGACGCAACGCGAAGAACCTTA
VCDY01000019.1_112-1667r      CACAAGCGGTGGAGCATGTGGTTTAATTCGACGCAACGCGAAGAACCTTA
VCDV01000019.1_2-1557         CACAAGCGGTGGAGCATGTGGTTTAATTCGACGCAACGCGAAGAACCTTA
VCDW01000017.1_112-1667       CACAAGCGGTGGAGCATGTGGTTTAATTCGACGCAACGCGAAGAACCTTA
LC133084.1                    --------------------------------------------------
LC133085.1                    --------------------------------------------------
LC133086.1                    --------------------------------------------------
LC133087.1                    --------------------------------------------------
```

```
X77849.1                        CCGGGGTTTGACATCCTGCGAACCTGGTGGAAACACTGGGGTGCC-TTCG
L09168.1                        CCGGGGTTTGACATCCTGCGAACCTGGTGGAAACACTGGGGTGCC-TTCG
CP017019.1_154745-156300        CCGGGGTTTGACATCCTGCGAACCTGGTGGAAACACTGGGGTGCCCTTCG
CP017019.1_147549-149104        CCGGGGTTTGACATCCTGCGAACCTGGTGGAAACACTGGGGTGCCCTTCG
CP017237.1_144877-146432        CCGGGGTTTGACATCCTGCGAACCTGGTGGAAACACTGGGGTGCCCTTCG
VCDX01000030.1_112-1667r        CCGGGGTTTGACATCCTGCGAACCTGGTGGAAACACTGGGGTGCCCTTCG
VCDY01000019.1_112-1667r        CCGGGGTTTGACATCCTGCGAACCTGGTGGAAACACTGGGGTGCCCTTCG
VCDV01000019.1_2-1557           CCGGGGTTTGACATCCTGCGAACCTGGTGGAAACACTGGGGTGCCCTTCG
VCDW01000017.1_112-1667         CCGGGGTTTGACATCCTGCGAACCTGGTGGAAACACTGGGGTGCCCTTCG
LC133084.1                      --------------------------------------------------
LC133085.1                      --------------------------------------------------
LC133086.1                      --------------------------------------------------
LC133087.1                      --------------------------------------------------

X77849.1                        GGGAACGCAGAGACAGGTGGTGCATNN--GTCGTCAGCTCGTGTCGTGAG
L09168.1                        GGGAACGCAGAGACAGGTGGTGCATGGTTGTCGTCAGCTCGTGTCGTGAG
CP017019.1_154745-156300        GGGAACGCAGAGACAGGTGGTGCATGGTTGTCGTCAGCTCGTGTCGTGAG
CP017019.1_147549-149104        GGGAACGCAGAGACAGGTGGTGCATGGTTGTCGTCAGCTCGTGTCGTGAG
CP017237.1_144877-146432        GGGAACGCAGAGACAGGTGGTGCATGGTTGTCGTCAGCTCGTGTCGTGAG
VCDX01000030.1_112-1667r        GGGAACGCAGAGACAGGTGGTGCATGGTTGTCGTCAGCTCGTGTCGTGAG
VCDY01000019.1_112-1667r        GGGAACGCAGAGACAGGTGGTGCATGGTTGTCGTCAGCTCGTGTCGTGAG
VCDV01000019.1_2-1557           GGGAACGCAGAGACAGGTGGTGCATGGTTGTCGTCAGCTCGTGTCGTGAG
VCDW01000017.1_112-1667         GGGAACGCAGAGACAGGTGGTGCATGGTTGTCGTCAGCTCGTGTCGTGAG
LC133084.1                      --------------------------------------------------
LC133085.1                      --------------------------------------------------
LC133086.1                      --------------------------------------------------
LC133087.1                      --------------------------------------------------

X77849.1                        ATGTTGGGTTAAGTCCCGCAACGAGNGCAACCCCTACCTTTAGTTGCCAG
L09168.1                        ATGTTGGGTTAAGTCCCGCAACGAGCGCAACCCCTACCTTTAGTTGCCAG
CP017019.1_154745-156300        ATGTTGGGTTAAGTCCCGCAACGAGCGCAACCCCTACCTTTAGTTGCCAG
CP017019.1_147549-149104        ATGTTGGGTTAAGTCCCGCAACGAGCGCAACCCCTACCTTTAGTTGCCAG
CP017237.1_144877-146432        ATGTTGGGTTAAGTCCCGCAACGAGCGCAACCCCTACCTTTAGTTGCCAG
VCDX01000030.1_112-1667r        ATGTTGGGTTAAGTCCCGCAACGAGCGCAACCCCTACCTTTAGTTGCCAG
VCDY01000019.1_112-1667r        ATGTTGGGTTAAGTCCCGCAACGAGCGCAACCCCTACCTTTAGTTGCCAG
VCDV01000019.1_2-1557           ATGTTGGGTTAAGTCCCGCAACGAGCGCAACCCCTACCTTTAGTTGCCAG
VCDW01000017.1_112-1667         ATGTTGGGTTAAGTCCCGCAACGAGCGCAACCCCTACCTTTAGTTGCCAG
LC133084.1                      --------------------------------------------------
LC133085.1                      --------------------------------------------------
LC133086.1                      --------------------------------------------------
LC133087.1                      --------------------------------------------------

X77849.1                        CGGGTAAAGCCGGGCACTCTAAAGGGACTGCCGGTGACAAACCGGAGGAA
L09168.1                        CGGGTAAAGCCGGGCACTCTAAAGGGACTGCCGGTGACAAACCGGAGGAA
CP017019.1_154745-156300        CGGGTAAAGCCGGGCACTCTAAAGGGACTGCCGGTGACAAACCGGAGGAA
CP017019.1_147549-149104        CGGGTAAAGCCGGGCACTCTAAAGGGACTGCCGGTGACAAACCGGAGGAA
CP017237.1_144877-146432        CGGGTAAAGCCGGGCACTCTAAAGGGACTGCCGGTGACAAACCGGAGGAA
VCDX01000030.1_112-1667r        CGGGTAAAGCCGGGCACTCTAAAGGGACTGCCGGTGACAAACCGGAGGAA
VCDY01000019.1_112-1667r        CGGGTAAAGCCGGGCACTCTAAAGGGACTGCCGGTGACAAACCGGAGGAA
VCDV01000019.1_2-1557           CGGGTAAAGCCGGGCACTCTAAAGGGACTGCCGGTGACAAACCGGAGGAA
VCDW01000017.1_112-1667         CGGGTAAAGCCGGGCACTCTAAAGGGACTGCCGGTGACAAACCGGAGGAA
LC133084.1                      --------------------------------------------------
LC133085.1                      --------------------------------------------------
LC133086.1                      --------------------------------------------------
LC133087.1                      --------------------------------------------------
```

```
X77849.1                      GGTGGGGATGACGTCAAATCATCATGCCCCTTATATCCCGGGCTACACAC
L09168.1                      GGTGGGGATGACGTCAAATCATCATGCCCCTTATATCCCGGGCTACACAC
CP017019.1_154745-156300      GGTGGGGATGACGTCAAATCATCATGCCCCTTATATCCCGGGCTACACAC
CP017019.1_147549-149104      GGTGGGGATGACGTCAAATCATCATGCCCCTTATATCCCGGGCTACACAC
CP017237.1_144877-146432      GGTGGGGATGACGTCAAATCATCATGCCCCTTATATCCCGGGCTACACAC
VCDX01000030.1_112-1667r      GGTGGGGATGACGTCAAATCATCATGCCCCTTATATCCCGGGCTACACAC
VCDY01000019.1_112-1667r      GGTGGGGATGACGTCAAATCATCATGCCCCTTATATCCCGGGCTACACAC
VCDV01000019.1_2-1557         GGTGGGGATGACGTCAAATCATCATGCCCCTTATATCCCGGGCTACACAC
VCDW01000017.1_112-1667       GGTGGGGATGACGTCAAATCATCATGCCCCTTATATCCCGGGCTACACAC
LC133084.1                    --------------------------------------------------
LC133085.1                    --------------------------------------------------
LC133086.1                    --------------------------------------------------
LC133087.1                    --------------------------------------------------

X77849.1                      GTGCTACAATGGCCTGTACAAAGGGGTGCGAAGGAGCGATCCGGAGCGAA
L09168.1                      GTGCTACAATGGCCTGTACAAAGGGGTGCGAAGGAGCGATCCGGAGCGAA
CP017019.1_154745-156300      GTGCTACAATGGCCTGTACAAAGGGGTGCGAAGGAGCGATCCGGAGCGAA
CP017019.1_147549-149104      GTGCTACAATGGCCTGTACAAAGGGGTGCGAAGGAGCGATCCGGAGCGAA
CP017237.1_144877-146432      GTGCTACAATGGCCTGTACAAAGGGGTGCGAAGGAGCGATCCGGAGCGAA
VCDX01000030.1_112-1667r      GTGCTACAATGGCCTGTACAAAGGGGTGCGAAGGAGCGATCCGGAGCGAA
VCDY01000019.1_112-1667r      GTGCTACAATGGCCTGTACAAAGGGGTGCGAAGGAGCGATCCGGAGCGAA
VCDV01000019.1_2-1557         GTGCTACAATGGCCTGTACAAAGGGGTGCGAAGGAGCGATCCGGAGCGAA
VCDW01000017.1_112-1667       GTGCTACAATGGCCTGTACAAAGGGGTGCGAAGGAGCGATCCGGAGCGAA
LC133084.1                    --------------------------------------------------
LC133085.1                    --------------------------------------------------
LC133086.1                    --------------------------------------------------
LC133087.1                    --------------------------------------------------

X77849.1                      TCCCAAAAAGCAGGTCTCAGTTCGGATTGCAGGCTGCAACTCGCCTGCAT
L09168.1                      TCCCAAAAAGCAGGTCTCAGTTCGGATTGCAGGCTGCAACTCGCCTGCAT
CP017019.1_154745-156300      TCCCAAAAAGCAGGTCTCAGTTCGGATTGCAGGCTGCAACTCGCCTGCAT
CP017019.1_147549-149104      TCCCAAAAAGCAGGTCTCAGTTCGGATTGCAGGCTGCAACTCGCCTGCAT
CP017237.1_144877-146432      TCCCAAAAAGCAGGTCTCAGTTCGGATTGCAGGCTGCAACTCGCCTGCAT
VCDX01000030.1_112-1667r      TCCCAAAAAGCAGGTCTCAGTTCGGATTGCAGGCTGCAACTCGCCTGCAT
VCDY01000019.1_112-1667r      TCCCAAAAAGCAGGTCTCAGTTCGGATTGCAGGCTGCAACTCGCCTGCAT
VCDV01000019.1_2-1557         TCCCAAAAAGCAGGTCTCAGTTCGGATTGCAGGCTGCAACTCGCCTGCAT
VCDW01000017.1_112-1667       TCCCAAAAAGCAGGTCTCAGTTCGGATTGCAGGCTGCAACTCGCCTGCAT
LC133084.1                    --------------------------------------------------
LC133085.1                    --------------------------------------------------
LC133086.1                    --------------------------------------------------
LC133087.1                    --------------------------------------------------

X77849.1                      GAAGTCGGAATCGCTAGTAATCGCGGATCAGCATGCCGCGGTGAATACGT
L09168.1                      GAAGTCGGAATCGCTAGTAATCGCGGATCAGCATGCCGCGGTGAATACGT
CP017019.1_154745-156300      GAAGTCGGAATCGCTAGTAATCGCGGATCAGCATGCCGCGGTGAATACGT
CP017019.1_147549-149104      GAAGTCGGAATCGCTAGTAATCGCGGATCAGCATGCCGCGGTGAATACGT
CP017237.1_144877-146432      GAAGTCGGAATCGCTAGTAATCGCGGATCAGCATGCCGCGGTGAATACGT
VCDX01000030.1_112-1667r      GAAGTCGGAATCGCTAGTAATCGCGGATCAGCATGCCGCGGTGAATACGT
VCDY01000019.1_112-1667r      GAAGTCGGAATCGCTAGTAATCGCGGATCAGCATGCCGCGGTGAATACGT
VCDV01000019.1_2-1557         GAAGTCGGAATCGCTAGTAATCGCGGATCAGCATGCCGCGGTGAATACGT
VCDW01000017.1_112-1667       GAAGTCGGAATCGCTAGTAATCGCGGATCAGCATGCCGCGGTGAATACGT
LC133084.1                    --------------------------------------------------
LC133085.1                    --------------------------------------------------
LC133086.1                    --------------------------------------------------
LC133087.1                    --------------------------------------------------
```

```
X77849.1                      TCCCGGGCCTTGTACACACCGCCCGTCACACCACGNNNNNTGGCAACACC
L09168.1                      TCCCGGGCCTTGTACACACCGCCCGTCACACCACGAAAGCTGGCAACACC
CP017019.1_154745-156300      TCCCGGGCCTTGTACACACCGCCCGTCACACCACGAAAGCTGGCAACACC
CP017019.1_147549-149104      TCCCGGGCCTTGTACACACCGCCCGTCACACCACGAAAGCTGGCAACACC
CP017237.1_144877-146432      TCCCGGGCCTTGTACACACCGCCCGTCACACCACGAAAGCTGGCAACACC
VCDX01000030.1_112-1667r      TCCCGGGCCTTGTACACACCGCCCGTCACACCACGAAAGCTGGCAACACC
VCDY01000019.1_112-1667r      TCCCGGGCCTTGTACACACCGCCCGTCACACCACGAAAGCTGGCAACACC
VCDV01000019.1_2-1557         TCCCGGGCCTTGTACACACCGCCCGTCACACCACGAAAGCTGGCAACACC
VCDW01000017.1_112-1667       TCCCGGGCCTTGTACACACCGCCCGTCACACCACGAAAGCTGGCAACACC
LC133084.1                    --------------------------------------------------
LC133085.1                    --------------------------------------------------
LC133086.1                    --------------------------------------------------
LC133087.1                    --------------------------------------------------

X77849.1                      CGAAGCCGGTGACCTNACCCGCGAGG-AAGGAGCCGTCTAAGGTGGGGCT
L09168.1                      CGAAGCCGGTGACCTAACCCGCGAGG-AAGGAGCCGTCTAAGGTGGGGCT
CP017019.1_154745-156300      CGAAGCCGGTGACCTAACCCGCGAGGGAAGGAGCCGTCTAAGGTGGGGCT
CP017019.1_147549-149104      CGAAGCCGGTGACCTAACCCGCGAGGGAAGGAGCCGTCTAAGGTGGGGCT
CP017237.1_144877-146432      CGAAGCCGGTGACCTAACCCGCGAGGGAAGGAGCCGTCTAAGGTGGGGCT
VCDX01000030.1_112-1667r      CGAAGCCGGTGACCTAACCCGCGAGGGAAGGAGCCGTCTAAGGTGGGGCT
VCDY01000019.1_112-1667r      CGAAGCCGGTGACCTAACCCGCGAGGGAAGGAGCCGTCTAAGGTGGGGCT
VCDV01000019.1_2-1557         CGAAGCCGGTGACCTAACCCGCGAGGGAAGGAGCCGTCTAAGGTGGGGCT
VCDW01000017.1_112-1667       CGAAGCCGGTGACCTAACCCGCGAGGGAAGGAGCCGTCTAAGGTGGGGCT
LC133084.1                    --------------------------------------------------
LC133085.1                    --------------------------------------------------
LC133086.1                    --------------------------------------------------
LC133087.1                    --------------------------------------------------

X77849.1                      GGTGATTGGGGTGAAGTCGTAACAAGGTAGCCGTATCGGAAGGTGCGGCT
L09168.1                      GGTGATTGGGGTGAAGTCGTAACAAGGTAGCCGTATCGGAAGGTGCGGCT
CP017019.1_154745-156300      GGTGATTGGGGTGAAGTCGTAACAAGGTAGCCGTATCGGAAGGTGCGGCT
CP017019.1_147549-149104      GGTGATTGGGGTGAAGTCGTAACAAGGTAGCCGTATCGGAAGGTGCGGCT
CP017237.1_144877-146432      GGTGATTGGGGTGAAGTCGTAACAAGGTAGCCGTATCGGAAGGTGCGGCT
VCDX01000030.1_112-1667r      GGTGATTGGGGTGAAGTCGTAACAAGGTAGCCGTATCGGAAGGTGCGGCT
VCDY01000019.1_112-1667r      GGTGATTGGGGTGAAGTCGTAACAAGGTAGCCGTATCGGAAGGTGCGGCT
VCDV01000019.1_2-1557         GGTGATTGGGGTGAAGTCGTAACAAGGTAGCCGTATCGGAAGGTGCGGCT
VCDW01000017.1_112-1667       GGTGATTGGGGTGAAGTCGTAACAAGGTAGCCGTATCGGAAGGTGCGGCT
LC133084.1                    --------------------------------------------------
LC133085.1                    --------------------------------------------------
LC133086.1                    --------------------------------------------------
LC133087.1                    --------------------------------------------------

X77849.1                      GGATCACCTCC
L09168.1                      GGATCACCTCC
CP017019.1_154745-156300      GGATCACCT--
CP017019.1_147549-149104      GGATCACCT--
CP017237.1_144877-146432      GGATCACCT--
VCDX01000030.1_112-1667r      GGATCACCT--
VCDY01000019.1_112-1667r      GGATCACCT--
VCDV01000019.1_2-1557         GGATCACCT--
VCDW01000017.1_112-1667       GGATCACCT--
LC133084.1                    -----------
LC133085.1                    -----------
LC133086.1                    -----------
LC133087.1                    -----------
```

Multiple 16S rRNA gene sequence alignments of the genes extracted from the genomes and the corresponding, previously PCR amplified genes of *Moorella thermoautotrophica* strains, including the representative partial 16S rRNA sequences deposited by Kimura et al. (2016) from OUT1-OTU4. Differences between X77849.1 and L09168.1 versus the majority of the other sequences are highlighted in light blue. Differences between the 16S rRNA gene sequences extracted from the genomes and the 16S rRNA genes representing OTU1 – OUT-4 of Kimura et al. (2016) are highlighted in yellow.
